# Mitofusin-Dependent ER Stress Triggers Glial Dysfunction and Nervous System Degeneration in a *Drosophila* Model of Friedreich’s Ataxia

**DOI:** 10.3389/fnmol.2018.00038

**Published:** 2018-03-06

**Authors:** Oliver Edenharter, Stephan Schneuwly, Juan A. Navarro

**Affiliations:** Department of Developmental Biology, Institute of Zoology, University of Regensburg, Regensburg, Germany

**Keywords:** Friedreich’s ataxia, *Drosophila melanogaster*, mitofusin, ER stress, glia, mitophagy, muscles, mtRosella

## Abstract

Friedreich’s ataxia (FRDA) is the most important recessive ataxia in the Caucasian population. It is caused by a deficit of the mitochondrial protein frataxin. Despite its pivotal effect on biosynthesis of iron-sulfur clusters and mitochondrial energy production, little is known about the influence of frataxin depletion on homeostasis of the cellular mitochondrial network. We have carried out a forward genetic screen to analyze genetic interactions between genes controlling mitochondrial homeostasis and *Drosophila* frataxin. Our screen has identified silencing of *Drosophila mitofusin* (*Marf*) as a suppressor of FRDA phenotypes in glia. *Drosophila Marf* is known to play crucial roles in mitochondrial fusion, mitochondrial degradation and in the interface between mitochondria and endoplasmic reticulum (ER). Thus, we have analyzed the effects of frataxin knockdown on mitochondrial morphology, mitophagy and ER function in our fly FRDA model using different histological and molecular markers such as tetramethylrhodamine, ethyl ester (TMRE), mitochondria-targeted GFP (mitoGFP), p62, ATG8a, LAMP1, Xbp1 and BiP/GRP78. Furthermore, we have generated the first *Drosophila* transgenic line containing the mtRosella construct under the UAS control to study the progression of the mitophagy process *in vivo*. Our results indicated that frataxin-deficiency had a small impact on mitochondrial morphology but enhanced mitochondrial clearance and altered the ER stress response in *Drosophila*. Remarkably, we demonstrate that downregulation of *Marf* suppresses ER stress in frataxin-deficient cells and this is sufficient to improve locomotor dysfunction, brain degeneration and lipid dyshomeostasis in our FRDA model. In agreement, chemical reduction of ER stress by means of two different compounds was sufficient to ameliorate the effects of frataxin deficiency in three different fly FRDA models. Altogether, our results strongly suggest that the protection mediated by *Marf* knockdown in glia is mainly linked to its role in the mitochondrial-ER tethering and not to mitochondrial dynamics or mitochondrial degradation and that ER stress is a novel and pivotal player in the progression and etiology of FRDA. This work might define a new pathological mechanism in FRDA, linking mitochondrial dysfunction due to frataxin deficiency and mitofusin-mediated ER stress, which might be responsible for characteristic cellular features of the disease and also suggests ER stress as a therapeutic target.

## Introduction

Friedreich’s ataxia (FRDA) is the most common autosomal recessive ataxia in the Caucasian population (Cossée et al., 1997). The presence of a pathological GAA expansion in the first intron of the human FXN *locus* impairs the correct transcription of the gene, leading to low levels of the protein frataxin and causing the disease (Campuzano et al., 1997). The function of frataxin as a cardinal regulator of the biosynthesis of iron-sulfur clusters seems to be well established (Tsai and Barondeau, 2010; Schmucker et al., 2011). However, recent studies have questioned this role (Moreno-Cermeño et al., 2013) and thus frataxin’s function is still a subject of controversy. Nevertheless, it has been demonstrated that reduced frataxin expression compromises the activity of iron-sulfur cluster-containing enzymes along with mitochondrial ATP production and mitochondrial membrane potential (Rötig et al., 1997; Lodi et al., 1999; Tan et al., 2001; Anderson et al., 2005; Shidara and Hollenbeck, 2010; Bolinches-Amorós et al., 2014).

The neurological pathology in FRDA is very prominent. Indeed, patients display several neurological phenotypes such as progressive degeneration of dorsal root ganglia (DRG), lesions in the dentate nucleus and corticospinal tracks as well as an atrophy of the peripheral nerves and a “dying back” neuropathy (reviewed in Koeppen and Mazurkiewicz, 2013). Although changes in other cell types are considered secondary, it is important to remark that tissues with an abundant expression of frataxin such as glial cells and skeletal muscles also display pathological manifestations. In this sense, a drastic demyelination of the large axons has also been reported in patients (Hughes et al., 1968; Koeppen et al., 2009; Koeppen and Mazurkiewicz, 2013; Corben et al., 2014), suggesting a critical dysfunction of Schwann cells. Strong loss of myelin, consistent with axonal defects, has also been described in rodent models (Simon et al., 2004; Al-Mahdawi et al., 2006). Similarly, targeted silencing of frataxin expression in glial cells in *Drosophila melanogaster* highlighted defects on lipid metabolism, oxidative stress and tissue integrity (Navarro et al., 2010). Moreover, frataxin depletion in different glial cell types resulted in a significant decrease in viability and proliferation, along with a boost of mitochondrial superoxide production, the activation of inflammatory and apoptotic responses and impairment of neuron-glia interactions (Lu et al., 2009; Loría and Díaz-Nido, 2015). Besides nervous system dysfunction, FRDA patients show a chronic myocarditis and a severe hypertropy of the myocardium (Russell, 1946; Weidemann et al., 2013; Koeppen et al., 2015) as well as progressive and symmetrical loss of muscle strength particularly affecting the lower limbs (Beauchamp et al., 1995). Magnetic resonance spectroscopy of the calf muscles in FRDA patients demonstrates impairment of ATP synthesis, inadequate oxygen utilization by muscles and prolonged recovery after exercise (Lynch et al., 2002; Nachbauer et al., 2012). In contrast, conditional ablation of frataxin in mouse muscles induces cardiac hypertrophy without relevant defects in skeletal muscles (Puccio et al., 2001).

Mitochondria are dynamic organelles that, depending on the physiological conditions, constantly undergo fusion and fission steps to supply the cell with ATP from oxidative phosphorylation (reviewed in Westermann, 2010, 2012). Therefore, it is reasonable to speculate that the mitochondrial dysfunction induced by RNAi mediated knockdown of frataxin would also affect the intracellular mitochondrial network altering the number, size and morphology of mitochondria, maybe further contributing to the pathogenesis of the disease. Indeed, it has been reported that frataxin knockdown in mammalian cells triggers the formation of giant or larger disorganized mitochondria in rodent muscles (Puccio et al., 2001) or in cultured cells (Calmels et al., 2009; Hick et al., 2013; Bolinches-Amorós et al., 2014; Obis et al., 2014). Other studies have also shown accumulation or hyperproliferation of mitochondria in mouse and cellular models (Payne et al., 2011; Hick et al., 2013, respectively). On the contrary, some reports have described increased mitochondrial fragmentation instead (Lefevre et al., 2012; Schiavi et al., 2015).

Notably, disruption of mitochondrial fusion and fission and mitochondrial quality control impairs normal distribution of mitochondria and in turn the local ATP production. This might be especially critical in neurons as they require large amounts of energy and depend mainly on mitochondrial respiration. Indeed, imbalance of mitochondrial dynamics has emerged as a common pathogenic element to several neurodegenerative diseases such as Parkinson’s disease, Charcot-Marie-Tooth, Alzheimer’s disease, Huntington’s disease or amyotrophic lateral sclerosis (reviewed in Reddy, 2014). In agreement, mitochondrial dynamics has been suggested as a possible target for therapeutic intervention in neurodegenerative disorders (Reddy, 2014; Civiletto et al., 2015). In the current working model of mitochondrial homeostasis, damaged or senescent mitochondria are detected when their inner membrane depolarizes. The decay in membrane potential activates a cellular pathway controlled by the Pink1 kinase and the ubiquitin-ligase Parkin that will modify the mitochondrial network by promoting the fusion of healthy mitochondria and the fission of inactive organelles prior to their degradation by mitophagy (reviewed in Westermann, 2010). This dynamic regulation, critical to mitochondrial function, relies on a core machinery formed by a group of GTPases named mitofusin (MFN1 and 2), optic atrophy 1 (OPA1) and dynamin-related protein 1 (DRP1) as well as on the mitochondrial quality control genes Pink1 and Parkin. The damaged organelles are finally degraded by macroautophagy in a process named mitophagy (Westermann, 2012). In recent years, several exciting studies have shown that the regulation of mitochondrial homeostasis is conserved in the fruit fly and that *Drosophila* is an excellent and pioneer model to analyze the leading role of mitochondrial dynamics in relation to neurodegenerative disorders (Corti et al., 2011). Most of the compelling evidences in the fruit fly regarding mitochondrial dynamics have come from studies in *Drosophila* adult indirect flight musculature and in dopaminergic neurons (Greene et al., 2003; Clark et al., 2006; Yang et al., 2006; Deng et al., 2008). Similarly, the core elements of mitophagy are also present in *Drosophila* (Vincow et al., 2013).

Despite the severe impact of frataxin depletion on mitochondria biology, to present, only two studies have addressed the impact of frataxin silencing on the homeostasis of the cellular mitochondrial network (Lefevre et al., 2012; Schiavi et al., 2015). Interestingly, frataxin silencing has also been reported to increase the expression or activity of endoplasmic reticulum (ER) stress markers (Cortopassi et al., 2006; Lu and Cortopassi, 2007; Huang et al., 2013) or to sensitize cells to chemically-induced ER stress (Cnop et al., 2012; Bolinches-Amorós et al., 2014). However, no direct mechanism linking ER with the pathology has yet been described in FRDA disease models.

In this work, we report the first genetic screen designed to analyze the influence of mitochondrial dynamics on FRDA phenotypes. Our results show that downregulation of *Mitofusin* is sufficient to rescue several defects triggered by frataxin deficiency in glia. *Mitofusin* has been shown to be involved in mitochondrial dynamics and mitochondrial-ER tethering in *Drosophila*. Our experiments dissecting the roles of *Mitofusin* in the fly FRDA model provide evidence that: (1) frataxin silencing induces accumulation of *Drosophila* p62 in muscles and glial cells; (2) the cellular machinery is able to detect the dysfunctional mitochondria and activates mitophagy for clearance of the damaged organelles; (3) the enhanced mitophagic flux is not sufficient to degrade the massive amount of affected mitochondria and this might trigger the accumulation of inefficient organelles and of autophagy markers; (4) additional activation of autophagy also might be beneficial; (5) frataxin knockdown enhances ER stress; (6) Mitofusin modulates the ER stress in frataxin-deficient glia and chemical reduction of ER stress improves several phenotypes in FRDA flies; and (7) for the first time in any FRDA model, we have been able to show that mitochondrial dysfunction and ER stress represent a crucial convergence point in the pathology of the disease.

## Materials and Methods

### *Drosophila* Stocks

Fly stocks were maintained at 25°C on standard cornmeal-agar medium (water: 36 l; yeast: 720 g; cornmeal: 3200 g; soy flour: 400 g; light malt extract: 3200 g; sugar beet syrup: 880 g; agar: 320 g, Nipagin 120 g). The crosses between the GAL4 drivers and the UAS responder lines were carried out at 25°C unless stated. UAS constructs were used in heterozygous configurations for all experiments. The stocks used in this work as well as their origin are listed in Table S1 of Datasheet 1 in the Supplementary Material. Genetic interactions were carried out by first generating the stocks *fhRNAi-1*/CyO; *Repo-GAL4*/TM6B *tub*-GAL80 and *fhRNAi-1*/CyO; *Mef2-GAL4*/TM6B *tub*-GAL80.

### Generation of mtRosella Transgenic Flies

cDNA for the *mtRosella* construct was kindly provided by Prof. Dr. Benedikt Westermann (Böckler and Westermann, 2014). A 1700 bp fragment was amplified using the primers mtRosellaFw (CTCGAGATGTGGACTCTCGGGCGCCG) and mtRosellaRv (GGTACCTCAAGCATCTTTTCCGGAATAGGCCAAG). The full length coding sequence of *mtRosella* was cloned into the Gateway^®^ entry vector pENTR/D-TOPO^®^ by BP-cloning (Invitrogen, Carlsbad, CA, USA) and was subcloned by LR-cloning (Invitrogen, Carlsbad, CA, USA) into PWH-attB plasmid vector (modified from PWH obtained from the *Drosophila* genomics Resource Center and kindly provided by Prof. Michael Krahn) generating the transgene UAS-mtRosella-PWH. The transgene was then integrated into the third chromosome of the *Drosophila* genome using the PhiC31 integrating cassette. Three independent lines were obtained for the construct.

### Real-Time PCR

Total RNA was extracted from 15 thoraces using peqGold TriFast reagent (PEQLAB Biotechnologie GMBH, Erlangen, Germany) following manufacturer’s instructions. Five-hundred nanogram mRNA were converted into cDNA using QuantiTect Rev. Transcription Kit (Qiagen GmbH, Hilden, Germany) and then used for qPCR with ORA qPCR Green ROX L Mix (HighQu, Kralchtal, Germany) on a CFX connect™ Real-Time PCR Detection System (Bio-rad, Hercules, CA, USA). The ribosomal protein 49 (*rp49*) was used as internal control. The results from at least four independent biological replicates were analyzed with the Bio-Rad CFX manager 3.1 software. Gene expression levels were referred to the internal control, the relative quantification was carried out by means of the ΔΔCt method and the results were plotted as relative mRNA expression. Each experiment consisted of 3–5 independent biological replicates. The genes and the pairs of primers used for the analysis are summarized in Table S2 of Datasheet 1 in the Supplementary Material.

### Analysis of Mitochondrial DNA (mtDNA) Levels

For DNA extraction, thoraces from 15 to 20 male flies of desired age were homogenized in 400 μl Buffer A (100 mM Tris, pH7.5, 100 mM EDTA, 100 mM NaCl, 0.5% SDS). After incubation at 65°C for 60 min with continuous shaking, 800 μl of LiCl/KAc (5 M KAc and 6 M LiCl in a relation 1:2.5) solution were added, followed by 10 min incubation on ice. After centrifugation (15 min, 13,000 rpm, RT), 1 ml supernatant was transferred in a new tube. The DNA was precipitated by adding 600 μl of isopropanole and vortexing. After centrifugation (15 min, 13,000 rpm, RT) the resulting pellet was washed with 70% ethanol and resuspended in 200 μl H_2_O. Isolated DNA was quantified by measuring the absorbance at 260 nm with a NanoDrop spectrophotometer (PEQLAB Biotechnologie GMBH, Erlangen, Germany) and frozen at −20°C. In order to estimate the amount of mtDNA, the levels of *mitochondrial cytochrome oxidase I* (Fw 5′-AACTGTTTACCCACCTTTATCTGCTB-3′ and Rv 5′-CCCGCTAAGTGTAAAGAAAAAATAGC-3′) were referred to the levels of the housekeeping gene *GAPDH* (Fw 5′-GACGAAATCAAGGCTAAGGTCG-3′ and Rv 5′-AATGGGTGTCGCTGAAGAAGTC-3′), using 10 ng of Template DNA per reaction. Reactions were performed with qPCR Green ROX L Mix (HighQu, Kralchtal, Germany) on a CFX connect™ Real-Time PCR Detection System (Bio-rad, Hercules, CA, USA). The relative quantification was carried out by means of the ΔΔCt method and the results were plotted as Relative mtDNA content. Each experiment consisted of 3–5 independent biological replicates.

### Western Blot

For Western blots, 15 larvae (L2 and L3), adult male flies or thoraces were homogenized in 80 μl cold RIPA buffer (Sigma-Aldrich, St. Louis, MO, USA) with complete protease inhibitor mixture (Roche, Mannheim, Germany). The protein levels were determined using the BCA protein quantification kit (Thermo Scientific, Schwerte, Germany). Samples were diluted 1:4 with standard 4× Laemmli buffer, boiled for 5 min and approximately 25–30 μg of proteins were separated on 5% stacking-10% separating SDS polyacrylamide gels. The resolved proteins were electroblotted to a Protran BA 85 nitrocellulose membrane (Hartenstein, Würzburg, Germany) and were probed using rabbit anti-p62 (1:3000, kindly provided by Gábor Juhász, Pircs et al., 2012), rabbit anti-phospho eIF2αS1 (1:1500, ab32157, abcam, Cambridge, UK) and rabbit anti-GFP (1:2500, A6455, Invitrogen, Carlsbad, CA, USA). Mouse anti-α-tubulin (1:5000, T9026, Sigma-Aldrich, St. Louis, MO, USA) and rabbit anti-actin (1:2500, Sigma-Aldrich, St. Louis, MO, USA) were used as loading controls. Secondary fluorescent goat anti-mouse 680 LT and goat anti-Rabbit 800 CW (1:10,000 and 1:5000 respectively, Li-Cor Inc., Bad Homburg, Germany) were used in all cases. Detection and quantification was conducted using the Odyssey system (Li-Cor Inc., Lincoln, NE, USA). Arbitrary fluorescent units were normalized to the internal loading control. Data were expressed as percentage of average control values and plotted as relative protein levels. At least three independent biological replicates were used for each genotype and condition. The original images of representative blots shown or quantified in this manuscript highlighting the area displayed in the corresponding figures are available in the Datasheet 2 in the Supplementary Material.

### Brain and Muscle Histology

Flies of appropriate age and genotype were fixed in 4% PFA for 2 h. Brains or thoraces were then dissected, washed with PBST and blocked with 10% Normal Goat Serum or 10% Normal Donkey Serum, depending on secondary antibody, and incubated 24 h at 4°C with the primary antibody: mouse anti-Repo (1:200, 8D12, Developmental Studies Hybridoma Bank, Iowa city, IA, USA), goat anti-GFP (1:100, 600-101-215, Rockland, PA, USA), rabbit anti-p62 (1:500, kindly provided by Sébastien Gaumer, Nezis et al., 2008) and rat anti-BiP (1:200, BT-GB-143P, Babraham Bioscience Technologies, Cambridge, UK). Then, samples were incubated 24 h at 4°C with secondary fluorescent antibodies: donkey anti-goat AF488, donkey anti-rabbit AF594, donkey anti-mouse AF555, goat anti-rabbit AF488, goat anti-rat AF555 (in all cases 1:200, Invitrogen, Carlsbad, CA, USA), goat anti-mouse Cy3 (1:200, Jackson Dianova, Hamburg, Germany), DAPI (10 μg/ml, Sigma-Aldrich, St. Louis, MO, USA) and TRITC-conjugated Phalloidin to visualize F-actin from muscle fibers (1:500, Sigma-Aldrich, St. Louis, MO, USA). Samples were embedded in Vectashield mounting medium (Vector Laboratories, Burlingame, CA, USA). At least 5–10 flies per genotype were scanned. GFP and antibody patterns were analyzed by scanning the samples using the multi-tracking setups of either a LSM 510 Confocal Microscope (Zeiss, Germany) or Confocal Laser Scanning Platform Leica TSC SP8 (Leica, Germany).

In order to analyze mitochondrial membrane potential in indirect flight muscles (IFM), flies were anesthetized with CO_2_ and the head, abdomen, wings and legs were removed. The thorax was cut sagitally with a sharp injector blade (Stainless Steel Injector Blades, Cat. #71990, Electron Microscopy Sciences, Hatfield, PA, USA). The hemithoraces were then incubated with 1 μm tetramethylrhodamine, ethyl ester (TMRE; Cat. #89917, Sigma-Aldrich, Germany) in Relaxing solution (20 mM KH_2_PO_4_, 5 mM MgCl_2_, 5 mM EGTA, 5 mM ATP) for 15 min, washed with Relaxing Solution three times for 5 min, mounted in VectaShield and immediately imaged.

All images were acquired using the same exposure, light intensity and filter settings for all conditions. Confocal images were further processed with the image processing software Fiji 2.0.0 (Schindelin et al., 2012). In detail, background was subtracted in ImageJ via the “Rolling Ball” method (Radius = 50 pixels). Maximum projections of three (muscle images) to 30 (brain images) slices were made and the resulting image was again subjected to background subtraction. Finally, contrast of each image was adjusted to improve quality of signal. Further details about confocal microscope settings and image handling are described in the Supplementary methods.

Paraffin sections were performed from 35-day-old adult flies. Flies were fixed with carnoy (ethanol:chloroform:acetic acid at a proportion 6:3:1), dehydrated in ethanol, and embedded in paraffin. Paraffin sections (7 μm) from 10 flies of each genotype were analyzed under a fluorescence microscope. Brain vacuolization was quantified using the ImageJ 1.48v software. The affected area was referred as % of total brain area that includes the lamina (La), the outer chiasm (OC) and the medulla (Me). For examination of lipid accumulation with light microscopy and analysis of degeneration of photoreceptor neurons, semithin epon plastic sections from 35-day-old and 5-day-old adult fly brains, respectively, were prepared. Lipids were identified by characteristically dark droplet-like structures.

### Aconitase Activity and ATP Quantity

Total aconitase activity was determined from whole individuals using Bioxytech Aconitase-340™ Spectrophotometric Assay kit (OXIS International Inc., Portland, OR, USA). Twenty-five adults or 15 thoraces were placed in a tube containing 500 μl of ice-cold homogenization buffer (0.2 mM sodium citrate and 50 mM Tris-HCl, pH 7.4) and gently pounded. The resulting mashes were centrifuged twice at 800 *g* for 10 min at 4°C discarding the pellet every time. Supernatants were disrupted by sonication for 30 s with 1 s interval between each pulse. One-hundred microliter of each extraction was used to measure the aconitase activity. The aconitase activity of each genotype was measured from four independent samples and each extraction was measured twice. The aconitase activity was finally referred to protein amount using the Bradford assay (Coomassie Plus™ Protein Assay Reagent, Thermo Scientific, Schwerte, Germany). ATP was determined using ATP Bioluminescence Assay Kit CLS II (Roche, Mannheim, Germany) according to manufacturer’s instructions with some modifications. Briefly, five thoraces were homogenized in 200 μl pre-heated ATP assay buffer (100 mM Tris, 4 mM EDTA, pH 7.75), boiled for 2 min and centrifuged for 1 min at 1000 *g*. The supernatant was diluted 1:3 in ATP assay buffer and 50 μl of the dilution were used to measure ATP levels. Luminescence was detected with a Tecan Infinite^®^ F500 plate reader (Tecan Trading AG, Switzerland). Each experiment consisted of 3–5 independent biological replicates.

### Lifespan, Chemical Treatments, Hyperoxia and Negative Geotaxis Assays

In all experiments (normal conditions and food supplementation), flies were raised at 25°C under a 12 h:12 h light/dark cycle and male individuals were collected within 24–48 h posteclosion, placed at a density of 25 per vial and transferred to vials with the corresponding fresh food every 2–3 days. Lifespan experiments were conducted in standard cornmeal agar medium and 100–150 males per genotype were used in each experiment. For chemical treatments, Instant *Drosophila* Medium (Formula 4–24, Carolina Biological, Whitsett, NC, USA) was rehydrated either with 15 mM Tauroursodeoxycholic acid (TUDCA, Merck Miilipore, MA, USA), 7.5 mM 4-Phenylbutyric acid (4-PBA, Sigma-Aldrich, St. Louis, MO, USA) or 50 μm Rapamycin (LC Laboratories, Woburn, MA, USA). As control treatment, Instant *Drosophila* Medium was reconstituted with distilled water or DMSO diluted in distilled water. Hyperoxia treatment started 1 day post-eclosion and was performed by exposing flies in a glass container with a constant flux of 99.5% oxygen under a low positive pressure at 25°C (Botella et al., 2004). Flies were transferred every day to new vials. Locomotor assays were performed as previously described (Botella et al., 2004). Ten to twelve flies per genotype were assessed and each fly was recorded three times and the mean value of each fly was used for the subsequent analysis.

### Statistical Analysis

Survival data were analyzed using the Log-rank (Mantel-Cox) and Gehan-Breslow-Wilcoxon tests. In all further experiments, data is represented as mean ± SEM of 4–5 replicates. When comparing two samples, equal variances were confirmed by an F test. When comparing multiple samples, equal variances were confirmed by Bartlett and Brown-Forsythe tests. Normality of data was assessed in all cases with Shapiro and Wilk test and parametric or non-parametric tests were used accordingly. For data that passed normality test, significance was determined by two-tailed *T*-test or by One-way ANOVA with *post hoc* Dunnett or Tukey Multiple Comparison Test (***P < 0.001; **P < 0.01 and *P < 0.05). Statistical analysis was carried out using Prism version 7.02 for Windows, GraphPad Software, La Jolla, CA, USA^1^.

### Preparation of Figures

All figures were assembled with Adobe Photoshop CC 2017.0.0 (Adobe Systems) by importing microscopy images from Fiji and graphs from Prism.

## Results

### Mitofusin Downregulation Rescues FRDA Glial Phenotypes

Downstream effects of frataxin downregulation such as altered transport, number, size and morphology of mitochondria (Puccio et al., 2001; Calmels et al., 2009; Shidara and Hollenbeck, 2010; Chen et al., 2016b) indicate defects in the stability, integrity and homeostasis of this organelle that might be contributing to the pathobiology of FRDA. However, the impact of mitochondrial dynamics on frataxin downregulation phenotypes has never been studied. This encouraged us to further investigate whether genetic manipulation of mitochondrial homeostasis might also provide beneficial effects in frataxin-deficient flies. We have reported previously that frataxin knockdown in glia affects fly locomotion, increases brain vacuolization due to cellular degeneration and triggers accumulation of lipids (Navarro et al., 2010). We now first assessed whether altered expression of genes involved in mitochondrial homeostasis had any influence on the locomotor performance of frataxin-deficient flies. Thus, *fhRNAi-1/CyO*; *Repo*-GAL4/TM6B *Tub*-GAL80 flies (Navarro et al., 2015) were mated with a collection of fly lines to increase or downregulate the expression of genes involved in mitochondrial fusion and fission (*Marf*, *Opa1* and *Drp1*), mitochondrial quality control (*Pink1* and *Parkin*) and mitochondrial biogenesis (*Spargel, Srl*). We decided to add a second group of genes involved in mitochondrial motility (*Miro*, *Milton* and *Khc*) since they have been shown to be important for glial function (Schmidt et al., 2012). Due to the known positive effects of antioxidant genes in FRDA flies (Anderson et al., 2008; Navarro et al., 2010), we also included in our analysis a constitutively expressed mitochondrial catalase (*mitocatalase*) construct as a secondary control of the reliability of our strategy. As expected, frataxin-deficient flies in glia showed reduced climbing ability (0.8 cm/s) compared to controls (1.5 cm/s). Importantly, coexpression of two neutral transgenes such as UAS-*GFP* and UAS-*mcherryRNAi* did not significantly alter the locomotion of FRDA flies. In addition, frataxin overexpression (UAS-*fh*) significantly rescued locomotor performance. As expected, expression of *mitocatalase* also ameliorated locomotor dysfunctions of frataxin-deficiency in glial cells (1.3 cm/s). All these results support the suitability of our experimental setup to test for genetic interactions.

**Figure 1:**
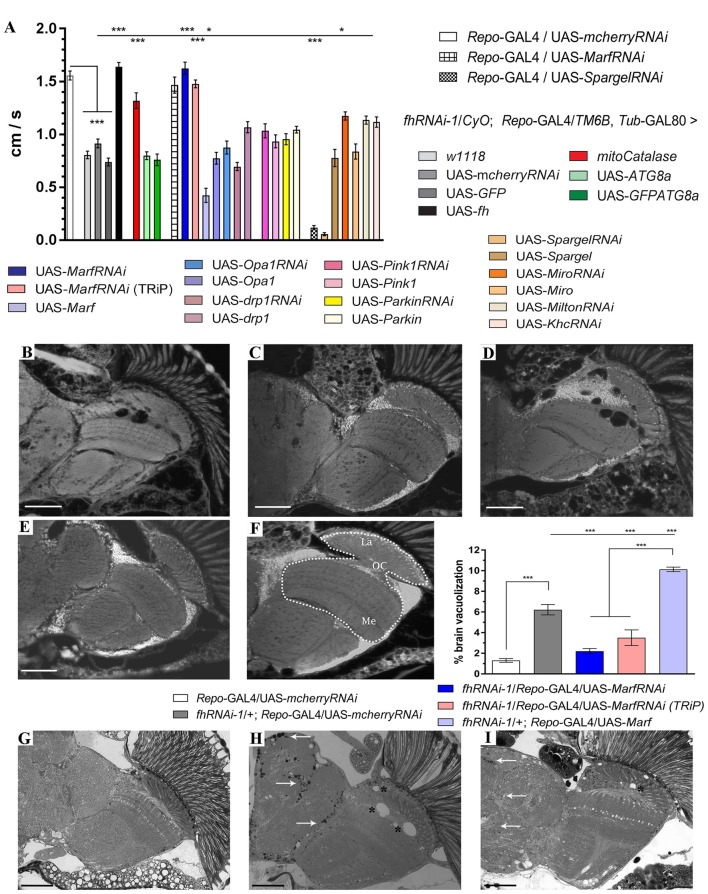
*Mitofusin* silencing improves locomotor ability, brain vacuolization and lipid accumulation in frataxin-deficient glial cells. **(A)** Negative geotaxis performance of 10-day-old flies displaying glial-specific frataxin silencing and coexpression of our transgene’s collection. **(B–E)** Representative brains from 35-day-old flies displaying frataxin knockdown in glial cells and (*fhRNAi-1*; *Repo*-GAL4) along with coexpression of **(B)** UAS-*mcherryRNAi*. A second transgene does not modify brain vacuolization and thus no GAL4 dilution effect is present, **(C)** UAS-*MarfRNAi*. Complete rescue of brain vacuolization and **(D)** UAS-*Marf*. Overexpression of Marf (MarfOE) results in a more severe brain vacuolization. **(E)** UAS-*MarfRNAi* (TRiP). A second RNAi line for *Marf* shows the same effect. **(F)** Wild type brain displaying the area of study (dotted white line) consisting of medulla, lamina and outer chiasm (OC). Quantification of degenerated area (%) in 35-day-old flies. Control flies show only minor vacuolization (1.3%) of the analyzed area. Frataxin deficiency increased the degeneration by five-fold which was suppressed by both *Marf* downregulations. *Marf* overexpression further enhanced the vacuolization. **(G)** Representative semithin plastic epon section of a 35-day-old control fly (*Repo*-GAL4/UAS-*mcherryRNAi*). No lipid accumulation and brain vacuolization was detected. **(H)** Representative semithin plastic epon section of a 35-day-old frataxin-deficient fly coexpressing a neutral transgene (*fhRNAi-1*/+; *Repo*-GAL4/UAS-*mcherryRNAi*). Strong lipid accumulation (arrows) and brain vacuolization (asterisks) were detected. **(I)** Representative semithin plastic epon section of a frataxin-deficient fly coexpressing a transgene to downregulate *Marf* (*fhRNAi-1*/+; *Repo*-GAL4/UAS-*MarfRNAi*). Clear reduction of the size of lipid droplets (arrows) and the number of brain vacuoles (asterisks) were observed. Graphs in **(A,F)** show means ± SEM. Data was analyzed by One-way ANOVA with *post hoc* Dunnett Multiple Comparison Test. *P < 0.05; ***P < 0.001. In all images, scale bar represents 50 μm.

Our results, summarized in Figure 1A, showed that among all lines tested only *Mitofusin* (*Marf*) downregulation, using an RNAi line extensively described in the literature (Deng et al., 2008; Dorn et al., 2011; Celardo et al., 2016), had a strong positive impact. *Marf* knockdown in glial cells rescued FRDA locomotion up to control levels (1.6 cm/s). Remarkably, compared to frataxin-deficient flies coexpressing the *mcherryRNAi* transgene (Figures 1B,H and quantification in Figure 1F), *Marf* silencing also suppressed brain vacuolization (Figure 1C and asterisks in Figure 1I and quantification in Figure 1F) and restored the lipid dysfunction as seen by the reduced number and size of lipid droplets in the fly brains (arrows in Figure 1I) with a similar aspect to controls (Figure 1G). In agreement, *Marf* overexpression worsened brain degeneration (Figure 1D and quantification in Figure 1F). A second RNAi line (TRiP) confirmed the ability of *Marf* silencing to rescue locomotor performance (Figure 1A) and the increased brain vacuolization (Figure 1E and quantification in Figure 1F) of frataxin deficiency.

Our results highlight *Marf* as a key molecule in the neurological dysfunction in FRDA.

### Accumulation of *Drosophila* p62 in Frataxin-Deficient Glial Cells

*Marf* has been extensively described as a mediator of mitochondrial fusion in mammals as well as in *Drosophila* (Westermann, 2010; Corti et al., 2011) and it has been reported to be a substrate of the ubiquitin ligase Parkin in the mitophagy process (Pallanck, 2013; Wang et al., 2016). Therefore, the genetic interaction with *Marf* downregulation might indicate alterations in mitochondrial morphology and degradation upon frataxin silencing in glial cells.

**Figure 2:**
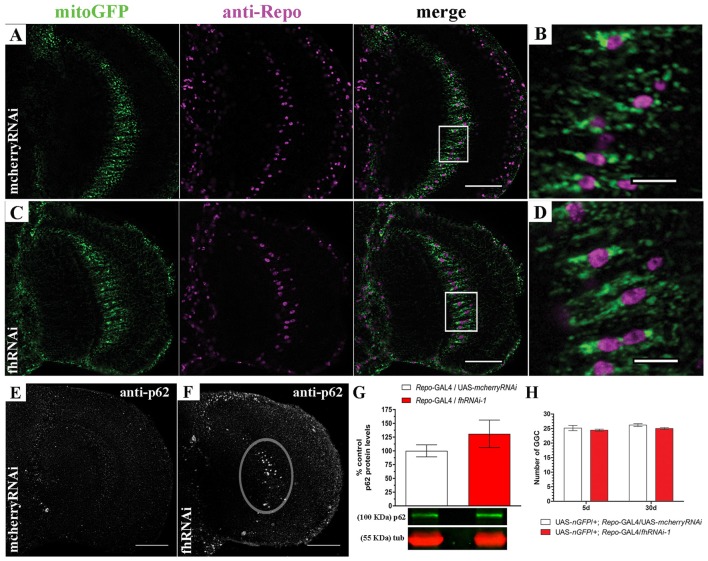
Targeted silencing of frataxin in glia alters mitochondrial homeostasis. **(A)** Representative optic lobe of a 35-day-old control fly (UAS-*mitoGFP*/+; *Repo*-GAL4/UAS-*mcherryRNAi*) stained with mitochondria-targeted GFP (mitoGFP) and the glial specific marker Repo. **(B)** Magnification of the giant glial cells (GGCs) in the inner optic chiasm from (**A**; white square). Mitochondria are broadly distributed. **(C)** Representative optic lobe of a 35-day-old Friedreich’s ataxia (FRDA) fly (UAS-*mitoGFP*/+; *Repo*-GAL4/*fhRNAi-1*). **(D)** Magnification of the GGCs in the inner optic chiasm from **(C**;white square). No evident alteration of mitochondrial distribution. **(E)** Optic lobe of a 35-day-old control stained with p62 antibody (UAS-*mitoGFP*/+; *Repo*-GAL4/UAS-*mcherryRNAi*). Presence of p62-positive signals throughout the brain due to the aging process (Nezis et al., 2008) without any accumulation in the inner optic chiasm. **(F)** Optic lobe of a 35-day-old FRDA fly (UAS-*mitoGFP*/+; *Repo*-GAL4/*fhRNAi-1*) stained with p62. Strong accumulation of p62 in the GGC cells (denoted area). **(G)** Western blot analysis of p62 levels in whole heads of control and frataxin deficient flies. No significant accumulation was detected. **(H)** Number of GGCs in control (UAS-*nGFP*/+; *Repo*-GAL4/UAS-*mcherryRNAi*) and frataxin-deficient (UAS-*nGFP*/+; *Repo*-GAL4/*fhRNAi-1*) flies. Only cells displaying nuclear GFP signal and repo immunoreactivity were used for quantification. No loss of cells was detected upon frataxin-silencing. Scale bars in **(A,C,E,F)** represent 40 μm and 10 μm for **(B,D)**. Graphs in **(G,H)** show means ± SEM. Statistical analysis was performed by two-tailed *T*-test **(G)** or one-way ANOVA with *post hoc* Dunnett Multiple Comparison test **(H)**.

In order to address this question, we decided to focus on one type of glial cell, the giant glial cells (GGCs) of the inner and outer optic chiasms on both sides of the medulla (Tix et al., 1997). These glial cells are a specialized form of ensheathing glia that wraps axonal tracts which project between neuropils. They are suitable for our analysis because of their large size and their location in the region that shows strong brain vacuolization upon frataxin silencing (Navarro et al., 2010). Mitochondria were visualized in glia by means of a mitochondria-targeted GFP (mitoGFP). The analysis of mitoGFP pattern between controls (Figures 2A,B; UAS-*mitoGFP*/+; *Repo*-GAL4/UAS-*mcherryRNAi*) and frataxin-deficient flies (Figures 2C,D; UAS-*mitoGFP*/+; *Repo*-GAL4/*fhRNAi-1*) did not reveal any remarkable difference in the distribution or morphology of mitoGFP signals throughout the glia. It is well known that reduction of frataxin expression affects mitochondrial function. However, the small cell size of glia and the structure of the *Drosophila* brain impedes the analysis of mitochondrial activity in our experimental scenario. Importantly, damaged mitochondria are normally degraded by a special form of autophagy called mitophagy (Westermann, 2010). Similarly to mammalian p62, the *Drosophila* ortholog Ref(2)P (hereafter referred to as p62) is also a classical marker of autophagic flux in the fly (Nezis et al., 2008; Pircs et al., 2012; Mauvezin et al., 2014). Interestingly, it has been recently reported that fly p62 might also be useful to monitor mitochondrial clearance by mitophagy upon organelle dysfunction (Wang et al., 2016). Reduction of frataxin expression in glial cells induced accumulation of p62 in our region of interest (denoted area, Figure 2F) compared to controls (Figure 2E). However, the difference could not be observed by western blot (Figure 2G), likely because of a general overall accumulation of p62 in aged brains (Nezis et al., 2008). Despite the accumulation of p62, targeted silencing of frataxin did not induce loss of GGCs (Figure 2H). Our results might suggest that frataxin deficiency in glial cells either blocks autophagy or increases the number of mitochondria labeled for degradation.

### Frataxin Depletion in *Drosophila* Muscle Cells Alters Mitochondrial Dynamics

**Figure 3:**
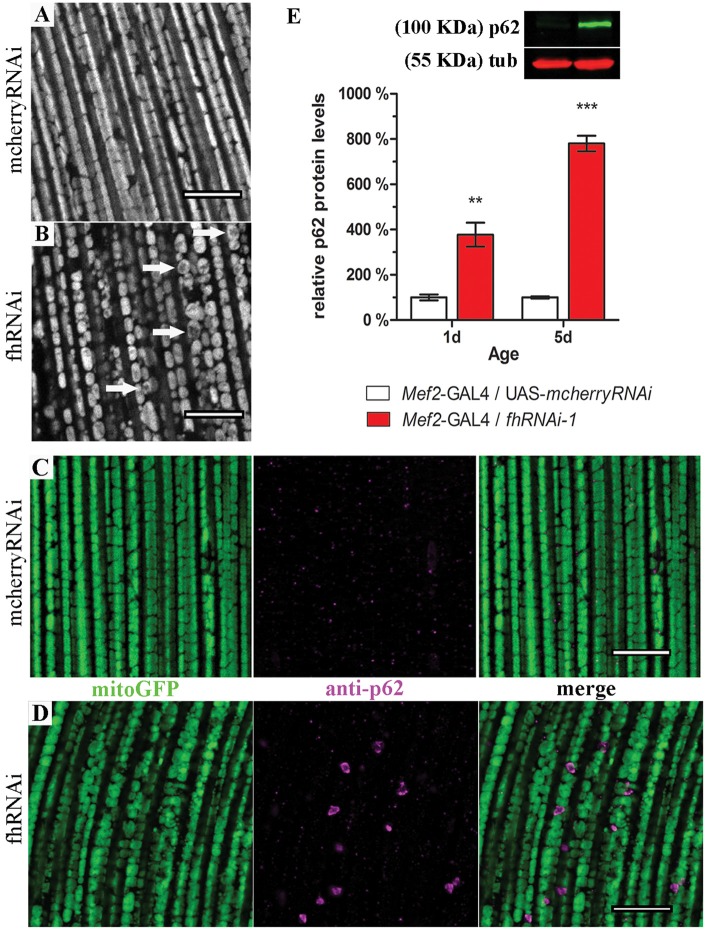
Frataxin silencing in muscles promotes mitochondrial fragmentation and accumulation of p62-positive structures. **(A,B)** MitoGFP labeled mitochondria from 10-day-old controls (**A**, UAS-*mitoGFP*/+; *Mef2*-GAL4/UAS-*mcherryRNAi*) and from 10-day-old frataxin-deficient thoraces (**B**, UAS-*mitoGFP*/+; *Mef2*-GAL4/*fhRNAi-1*). **(B)** A strong fragmentation was detected. Arrows indicate donut-like mitochondria in which the GFP signal is severely reduced as an indication of disorganized cristae. **(C)** *Drosophila* p62 staining in 5-day-old control muscles (UAS-*mitoGFP*/+; *Mef2*-GAL4/UAS-*mcherryRNAi*). **(D)** *Drosophila* p62 staining in 5-day-old frataxin-deficient muscles (UAS-*mitoGFP*/+; *Mef2*-GAL4/*fhRNAi-1*). Several p62 positive signals were detected. **(E)** Representative example of a p62 Western blot and quantification of p62 protein levels in FRDA flies 1 day and 5 days posteclosion compared to age-matched controls. Strong accumulation of p62 upon frataxin silencing in *Drosophila* muscles. Scale bar represents 10 μm in **(A–D)**. Graph in **(E)** represents means ± SEM. Statistical analysis was performed by Unpaired *T*-test. **P < 0.01; ***P < 0.001.

In order to address this question, we expanded our analysis to indirect flight musculature (hereafter referred to as muscles). *Drosophila* muscles comprise a large number of mitochondria and therefore, this tissue is extremely sensitive to mitochondrial perturbations which can be easily visualized and studied by histological approaches (Greene et al., 2003; Clark et al., 2006; Deng et al., 2008). By means of two different RNAi lines, our results demonstrate that frataxin knockdown in muscles reproduces the main features of the disease including reduced longevity and locomotion and impaired aconitase activity, ATP production and mitochondrial membrane potential (Supplementary Figure S1) in agreement with Lodi et al. (1999) and Vorgerd et al. (2000). Compared to controls (Supplementary Figures S2A, 3A), we also found that mitochondria in frataxin-deficient muscles display an age-dependent mitochondrial fragmentation from day 1 on (normal morphology, Supplementary Figure S2B) to day 10 (fragmented, Figure 3B). In line with the fragmentation, analysis of the expression profile of genes involved in mitochondrial dynamics and quality control showed important alterations (Supplementary Figure S2E). Interestingly, an increased mitochondrial fragmentation, as we have observed in frataxin-deficient muscles, is consistent with mitochondria being targeted for clearance by mitophagy (Palikaras and Tavernarakis, 2014). Indeed, frataxin-deficient muscles displayed a clear increase of p62 positive signals (*Mef2*-GAL4/*fhRNAi-1*; Figure 3D) compared to controls (*Mef2*-GAL4/UAS-*mcherryRNAi*; Figure 3C). Western blot analysis also showed that p62-levels were 4–8 times higher in frataxin-deficient flies than in controls and that they increased by 2-fold between 1-day-old and 10-day-old flies (Figure 3E). This was further confirmed by the analysis of number and size of p62-positive signals at three different ages (Supplementary Figures S2H–J). Interestingly, our results also show that mitochondrial dysfunction preceeds the organelle fragmentation since p62 starts to accumulate and mitochondrial TMRE signal is already decreased in 1-day-old flies (Figure 3E and Supplementary Figure S1L, respectively). Remarkably, p62 formed vesicle-like structures that are engulfing damaged mitochondria for degradation (Figure 3D and Supplementary Figure S2L). In most of the cases, similar results were observed with a second independent RNAi line for frataxin (fhRNAi-2, Supplementary Figure S2). Furthermore, mitochondrial DNA (mtDNA) content positively paralleled p62 levels (Supplementary Figure S2M) suggesting a clear mitochondrial accumulation. Our results show that frataxin deficiency in muscles alters mitochondrial dynamics and confirms that frataxin downregulation in the fly triggers accumulation of p62. Interestingly, the formation of p62-positive vesicles suggests a specific effect on mitophagy rather than a general autophagy dysfunction.

### Mitophagy Is Not Impaired in Frataxin-Deficient Cells

**Figure 4:**
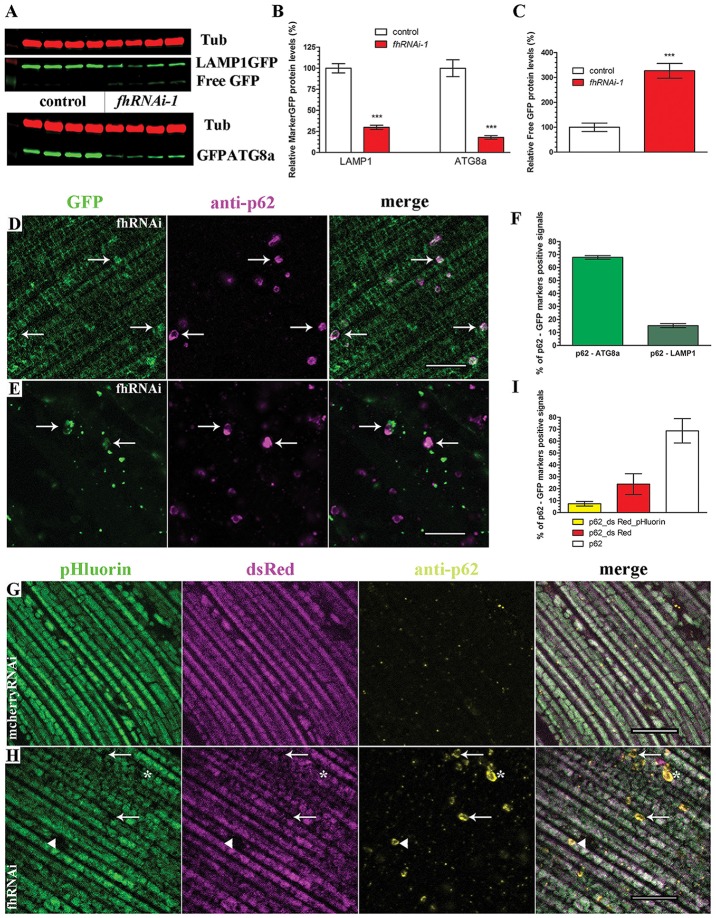
Enhanced mitophagic flux in frataxin-deficient cells. **(A)** Western blots showing levels of LAMP1GFP and GFPATG8a markers as well as Free GFP levels in controls and FRDA brain samples. **(B,C)** Quantification of blot in **(A)**. Strong reduction of protein levels of LAMP1GFP and GFPATG8a markers and increased levels of Free GFP upon frataxin silencing in glia cells. These results suggest increased mitophagic flux in glia. **(D)** Representative picture of LAMP1GFP and p62 patterns in 5-day-old frataxin-deficient flies (UAS-*LAMP1GFP*/+; *Mef2*-GAL4/*fhRNAi-1*). Increased punctuate structures. Arrows denote colocalization of both signals. **(E)** Representative picture of GFPATG8a and p62 patterns in 5-day-old frataxin-deficient flies *(fhRNAi-1*/+; *Mef2*-GAL4/UAS-*GFPATG8a*). Arrows denote colocalization of both signals. **(F)** Quantification of colocalization between p62 and GFPATG8a and LAMP1GFP signals referred to the total amount of p62 vesicles. Correct recruitment of autophagic machinery. **(G)** Representative picture of mtRosella and p62 patterns in 5-day-old control flies (*Mef2*-GAL4/*UAS-mtRosella*). Presence of both mtRosella signals in all mitochondria and absence of p62 signals. **(H)** Representative picture of mtRosella and p62 patterns in 5-day-old frataxin-deficient flies *(fhRNAi-1*/+; *Mef2*-GAL4/UAS-*mtRosella*). Presence of both signals in all mitochondria that have not been targeted for clearance (p62 negative). Arrows denote engulfed mitochondria that have lost dsRed and pHluorin signals. Asterisks denote mitochondria that only retained dsRed signal indicating location in the lysosomal compartment. A small population of p62-positive mitochondria still showed both mtRosella signals (arrow head). **(I)** Evaluation of colocalization between p62 and mtRosella signals referred to the total amount of p62 vesicles. Reduced percentage of mitochondria displaying all three signals (p62 and dsRed/pHluorin from mtRosella) whereas the majority showed only the pH-resistant dsRed or complete loss of any mtRosella signal, indicating correct clearance of defective mitochondria. Scale bars represent 10 μm in all cases. All graphs represent means ± SEM. Statistical analysis in **(B,C)** was performed by Unpaired *T*-test. ***P < 0.001.

**Figure 5:**
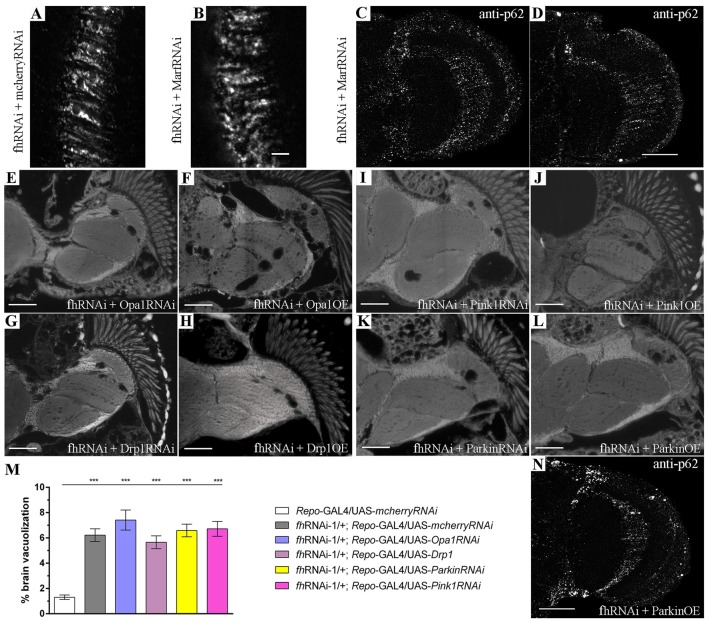
*Marf* silencing neither modifies the mitochondrial network nor improves mitochondrial degradation in FRDA glia. **(A,B)** Representative pictures of mitoGFP-labeled mitochondria from GGCs in 35-day-old frataxin-deficient (**A**, *fhRNAi-1*/UAS-*mitoGFP*; *Repo*-GAL4/UAS-*mcherryRNAi*) and frataxin-deficient coexpressing *Marf* silencing (**B**, *fhRNAi-1*/UAS-*mitoGFP*; *Repo*-GAL4/UAS-*MarfRNAi*) flies. Mitofusin downregulation did not alter the mitochondrial morphology in frataxin-deficient glial cells. **(C,D)** Examples of p62 staining of 35-day-old frataxin-deficient GGCs coexpressing UAS-*MarfRNAi* (*fhRNAi-1*/+; *Repo*-GAL4/UAS-*MarfRNAi*). The presence of p62-positive structures suggests that improvement of FRDA phenotypes is not a consequence of enhancing mitochondrial function or stimulation of mitophagy. **(E–L)** Representative images of brains from 35-day-old flies displaying frataxin knockdown in glial cells (*fhRNAi-1*; *Repo*-GAL4) along with coexpression of UAS-*Opa1RNAi* **(E)**, UAS-*Opa1* **(F)**, UAS-*Drp1RNAi* **(G)**, UAS-*Drp1* **(H)**, UAS-*Pink1RNAi* **(I)**, UAS-*Pink1* **(J)**, UAS*-ParkinRNAi* **(K)** and UAS-*Parkin* **(L)**. **(M)** Quantification of degenerated area (%) in 35-day-old frataxin-deficient flies. Genetic manipulation of mitochondrial fusion and fission or mitochondrial quality controls did not rescue brain vacuolization. **(N)** p62 staining of 35-day-old frataxin-deficient GGCs coexpressing UAS-*Parkin* (*fhRNAi-1*/ UAS-*Parkin*; *Repo*-GAL4/+). Induction of mitophagy by parkin overexpression did not improve accumulation of p62. Scale bars represent 40 μm in **(A–D,N)** and 50 μm in the other panels. Graph in **(M)** represents means ± SEM. Statistical analysis was performed by One-way ANOVA with *post hoc* Dunnett Multiple Comparison test. ***P < 0.001.

On the one hand, the detection of p62-positive vesicles indicates that the mitochondrial quality control mechanism driven by *pink1* and *parkin* is working properly in frataxin-deficient flies. On the other hand, accumulation of p62 inversely correlates with autophagic degradation (Bartlett et al., 2011; Pircs et al., 2012) and might indicate a failure of the autophagosome-lysosomal pathway. Therefore, we decided to study the autophagic flux in frataxin-deficient cells analyzing the amount and cellular distribution of two autophagy markers, LAMP1GFP and GFPATG8a, in both tissues (Figure 4). Our Western Blot analysis in glial cells (Figures 4A,B) showed that the levels of both proteins were decreased by around 80% in frataxin-deficient brains. Accordingly, levels of free GFP were increased (Figure 4C). Appearance of cleaved GFP has been previously linked to an efficient flux (Mauvezin et al., 2014; Bargiela et al., 2015). These results suggested an increased mitophagic flux. The small size of lysosomes, autophagosomes and mitochondria in glia (Supplementary Figures S3A–D and Figure 5A) prevented a reliable and robust histological analysis to confirm our findings. We then decided to address this question in the fly muscles. Compared to controls (Supplementary Figures S3F,G), we found a strong accumulation of punctate signals of both autophagy markers in frataxin-deficient muscles (arrows in Figures 4D,E) along with a partial colocalization of p62 with LAMP1GFP or GFPATG8a (Figure 4F, 20% and 70%, respectively). This result indicated a correct recruitment of the autophagic machinery to form autophagosomes (GFPATG8a), although formation of the autophagolysosomes might be a limiting step (LAMP1GFP). Similarly to glial cells, molecular analysis in muscles also suggested enhanced cleavage and turnover of LAMP1GFP, albeit to a lesser extent (Supplementary Figure S3E). In order to study whether the problem may lie in the inability to complete the degradation of the mitochondria, we have generated transgenic flies to express the mtRosella transgene and perform analysis of mitophagy *in vivo*. This construct consists of a mitochondria-targeted dsRed fluorescent protein fused to a pH-sensitive GFP named pHluorin. Under normal conditions, the green and red signals overlap in mitochondria, whereas, upon mitophagy, degenerating mitochondria are transferred to lysosomal compartments and the green signal is lost due to the low pH (Rosado et al., 2008). We have observed that, in controls, all mitochondria clearly showed both signals (Figure 4G). In frataxin-deficient flies, dysfunctional mitochondria were first marked with p62, which still showed both mtRosella signals (arrow heads in Figure 4H). Then, damaged organelles were incorporated into the autophagosome and, after fusion with lysosomes, the acidic pH eliminated the green sensitive signal from the pHluorin (asterisks in Figure 4H). Finally, the process of mitochondrial clearance was completed and the red signal from mtRosella marker was also depleted (arrows in Figure 4H). As expected, all mitochondria that were not enveloped by p62-vesicles displayed both signals. Quantification of the population of mitochondria that have entered the mitophagy process (p62-positive mitochondria) showed three groups of organelles: a reduced fraction (7%) displayed both signals (Figure 4I, yellow bar), a substantial 23% only displayed dsRed indicating incorporation into lysosomal compartment (Figure 4I, red bar) and importantly, the majority (70%) of the labeled mitochondria seemed to have lost both mtRosella signals (Figure 4I, white bar).

Altogether, our results clearly exclude mitophagy dysfunction in frataxin-deficient flies and thus, accumulation of p62 may suggest that the normal cellular capacity is insufficient to deal with the increasing number of damaged mitochondria. Therefore, we tested whether promotion of autophagy might improve FRDA conditions. Expression of the autophagy marker GFPATG8a in glial cells was accompanied by a clear reduction of p62 levels (Supplementary Figure S4A) and of brain vacuolization (Supplementary Figures S4B,D) compared to the expression of LAMP1GFP (Supplementary Figures S3B, S4C,D, respectively). Since it might be controversial whether GFPATG8a is a functional protein, we repeated the experiment using an independent UAS-ATG8a line and reduction of p62 levels and brain vacuolization were also observed (Supplementary Figures S4D–F). The effect of genetic autophagy induction was also detected in muscles. Our experiments revealed that overexpression of GFPATG8a triggered a strong reduction of p62 levels (40%) in FRDA muscles compared to the expression of two other GFP markers such as LAMP1GFP and ERGFP (Supplementary Figure S4G). However, chemical induction of early autophagy by means of rapamycin failed to improve locomotion and brain degeneration upon depletion of frataxin in glia (Supplementary Figures S4H,I).

### *Marf* Silencing Fails to Improve Mitochondrial Homeostasis in Glial Cells

Since promotion of autophagy had a positive impact on FRDA phenotypes, we decided to analyze whether the rescue of frataxin-deficiency, which is triggered by *Marf* downregulation, was due to the enhancement of mitochondrial clearance or to any additional modification of the mitochondrial network. *Marf* knockdown in frataxin-deficient glial cells (Figure 5B) did not produce any visible effect on mitochondrial morphology and distribution compared to frataxin-deficient glia (Figure 5A). In agreement, neither reduction of *Opa1* (*Marf* partner in mitochondrial fusion) nor overexpression of *Drp1* (fission mediator) were able to ameliorate the locomotor dysfunction (Figure 1A) and the brain vacuolization triggered by glial frataxin deficiency (Figures 5E–H and quantification in Figure 5M). These results suggest that fusion and fission of mitochondria are not the driving force underlying mitofusin-mediated rescue. Next, we tested whether *Marf* silencing was able to improve autophagic flux and to reduce p62 accumulation. *Marf* knockdown in frataxin-deficient glial cells failed to reduce p62 levels (Figures 5C,D). In this line of findings, suppression or overexpression of the mitochondrial quality control genes *Pink1* and *Parkin* (master regulators of mitophagy) failed to ameliorate any frataxin-deficient phenotypes such as negative geotaxis (Figure 1A), brain degeneration (Figures 5I–L and quantification in Figure 5M) and p62 accumulation (Figure 5N).

Remarkably, these results suggest that *Marf* silencing rescued FRDA phenotypes without improving mitochondrial function and that the protection mediated by *Marf* inhibition is mainly independent of *Marf* roles in mitochondrial fusion and clearance by mitophagy.

### Altered Endoplasmic Reticulum Stress Reponse in FRDA Flies

In mammalian cells, mitofusins are also involved in the interface between mitochondria and endoplasmic reticulum (ER). These connections facilitate the exchange of lipids and calcium and promote mitochondrial fission (van Vliet et al., 2014). Interestingly, *Marf* downregulation has been shown to induce ER stress in *Drosophila* muscles (Debattisti et al., 2014). ER stress activates in turn the unfolded protein response (UPR). Such a response is mediated by pancreatic ER kinase (PERK), inositol-requiring enzyme 1 (IRE1) and activating transcription factor 6 (ATF6), which trigger three cascades characterized by the increased expression of downstream transcription factors named ATF4, Xbp1 and the chaperone BiP/GRP78 (reviewed in Hetz and Mollereau, 2014). Importantly, the UPR is conserved in *Drosophila* and has already been associated with some fly models of neurological disorders (reviewed in Ryoo, 2015). This motivated us to determine whether frataxin silencing in flies was also affecting the ER morphology and stress.

**Figure 6:**
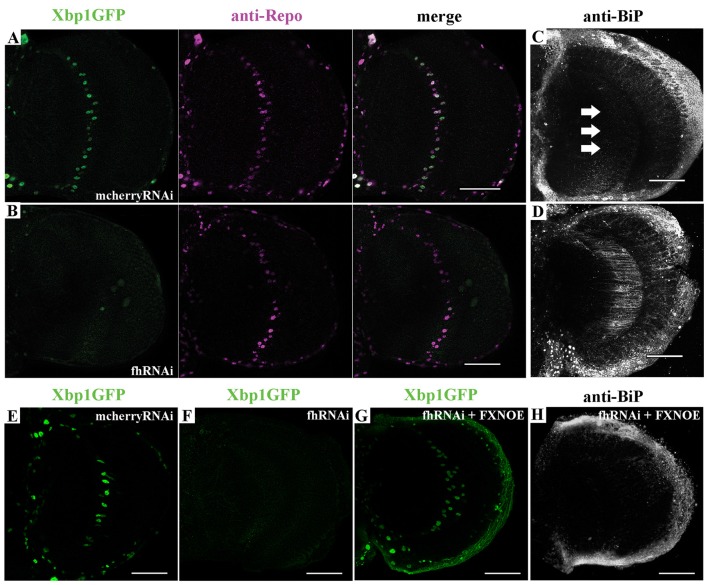
Frataxin depletion alters endoplasmic reticulum (ER) stress response in glia. **(A)** Representative image of 35-day-old control GGCs (UAS-*Xbp1GFP*/+; *Repo*-GAL4/+) stained with anti-Repo antibody to label cell nuclei. Strong and panglial induction of spliced Xbp1 that localizes in the cell nuclei. **(B)** Representative image of 35-day-old frataxin-deficient GGCs (*fhRNAi-1*/UAS-*Xbp1GFP*; *Repo*-GAL4/+). Induction of spliced Xbp1 was completely abolished. **(C)** Representative image of 35-day-old control GGCs (*Repo*-GAL4/UAS-*mcherryRNAi*) stained with anti-BiP to monitor ER stress levels. No induction of BiP. Arrows denote the area of interest. **(D)** Representative image of 35-day-old frataxin-deficient GGCs (*Repo*-GAL4/*fhRNAi-1*) stained with anti-BiP. Strong activation of ER stress response via induction of BiP levels. **(E,F)** Lack of Xbp1GFP induction already detected in young (7-day-old) frataxin deficient flies **(F)** compared to age-matched control **(E)**. **(G,H)** Coexpression of human frataxin (FXN) restores the nuclear localization of *Xbp1GFP* **(G)** and BiP expression **(H)** to wild type levels. Scale bars represent 40 μm in all cases.

**Figure 7:**
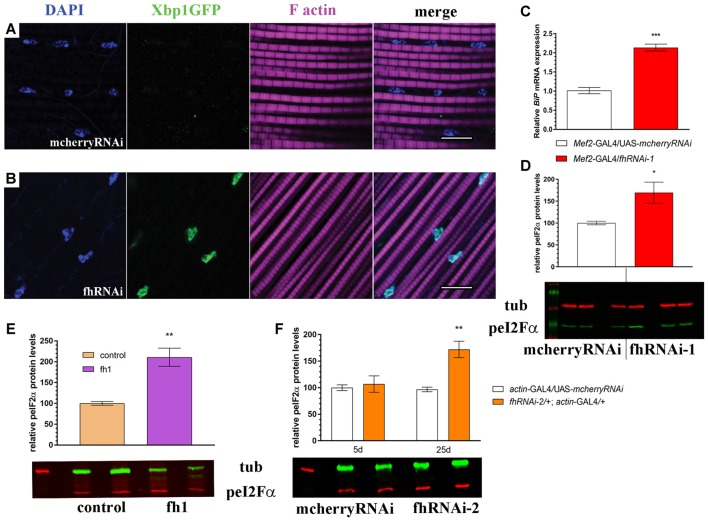
Muscle-specific and ubiquitous reduction of frataxin function triggers ER stress. **(A)** Representative image of 7-day-old control muscles (UAS-*Xbp1GFP*/+; *Mef2*-GAL4/+) stained with DAPI to label cell nuclei and phalloidin to label F-actin in muscle fibers. No induction of spliced Xbp1 was observed. **(B)** Representative image of 7-day-old frataxin-depleted muscles (UAS-*Xbp1GFP*/*fhRNAi-1*; *Mef2*-GAL4/+) stained with DAPI to label cell nuclei and phalloidin to label to label F-actin in muscle fibers. Strong induction of spliced Xbp1 was detected in cell nuclei. **(C)** Relative expression levels of *BiP/GRP78* in muscles of 7-day-old controls (*Mef2*-GAL4/UAS*-mcherryRNAi*) and frataxin-deficient muscles (*Mef2*-GAL4/*fhRNAi-1*). Robust (2-fold) activation of *BiP/GRP78* in FRDA flies. **(D)** Increased phosphorylation levels of eI2Fα in frataxin-deficient muscles. All these results indicate activation of all three branches from the unfolded protein response (UPR) and the boost of ER stress response in FRDA muscles. **(E,F)** Quantification of phosphorylation levels of eI2Fα in two additional models of FRDA. **(E)** A mutant allele of frataxin (fh1) and **(F)** a second RNAi line (fhRNAi-2). Significantly increased phosphorylation is detected in both cases, although only in old flies, when fhRNAi-2 was used. Scale bars represent 10 μm in all cases. Data in **(C–F)** were analyzed by Unpaired *T*-test. Means ± SEM. *P < 0.05; **P < 0.01; ***P < 0.001.

First, we tested *in vivo* in 35-day-old brains whether frataxin-deficiency in glia cells induced some markers of ER stress such as increased levels of the protein BiP and of a Xbp1GFP reporter, which is only expressed in-frame with GFP when IRE1-mediated splicing of Xbp1 occurs (Sone et al., 2013). Surprisingly, we detected that spliced Xbp1 was constitutively expressed in nuclei of control cells (Figure 6A), whereas its expression was completely abolished upon frataxin deficiency (Figure 6B). The lack of Xbp1 induction in FRDA glial cells was already evident in young flies (Figure 6F) compared to age-matched controls (Figure 6E). Interestingly, we found that FRDA flies exhibited increased BiP immunoreactivity (Figure 6D) compared to controls (Figure 6C) in the region of interest (arrows in Figure 6C). Activation of ER stress pathways in the glia was not accompanied with evident morphological defects in the ER (Supplementary Figures S5A,B). In order to exclude the influence of off-targets in the ER stress response observed upon targeted silencing of frataxin in glia, altered levels of Xbp1 reporter as well as chaperone BiP were analyzed after coexpression of human frataxin (UAS-*FXN*). Human frataxin is insensitive to RNAi contructs against fly frataxin and has already improved frataxin-deficient conditions in other tissues (Navarro et al., 2011; Tricoire et al., 2014). As expected, expression of FXN restored Xbp1 expression (Figure 6G) and reduced BiP levels (Figure 6H) in frataxin-deficient glia. The altered ER stress response was not restricted to glial cells. Frataxin knockdown in muscles also triggered the activation of all three UPR branches (Figures 7A–D). In this case, frataxin-deficient muscles displayed a strong nuclear signal of the Xbp1 reporter, whereas controls did not show any activation (Figures 7A,B). Frataxin-deficient muscles also showed elevated levels of BiP (Figure 7C) and of phosphorylated α subunit of eukaryotic translation initiation factor 2 (eIF2α; Figure 7D), a known marker of ER stress via PERK signaling (Celardo et al., 2016). An increased clustering of ER cisternae (Supplementary Figures S5C,D) was also detected in FRDA muscles. Moreover, we also took advantage of a severe loss-of-function allele of fly frataxin (fh1, Chen et al., 2016b) and of a second RNAi line that decreases frataxin expression to levels similar to those observed in patients (*fhRNAi-2*, Llorens et al., 2007). In both cases (Figures 7E,F), severe and moderate ubiquitous loss of frataxin also increased the levels of phosphorylated eIF2α. However, since phospho-eIF2α levels can be also increased by other pathways (Baker et al., 2012), we cannot completely rule out that other mitochondrial stress responses are also influencing the increase detected in fh1 and fhRNAi-2 flies. These results clearly suggest that frataxin-depletion dramatically alters the ER stress response ubiquitously and specifically in glia and muscles.

### Endoplasmic Reticulum Stress Is an Instrumental Pathological Element in FRDA

**Figure 8:**
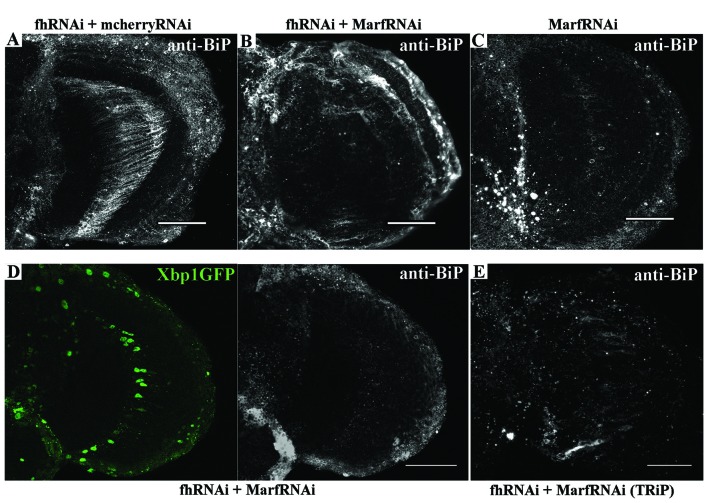
Marf regulates ER stress in frataxin deficient glial cells. **(A)** Representative image of 35-day-old frataxin-deficient GGCs coexpressing a control construct (*fhRNAi-1*/+; Repo-GAL4/ UAS-*mcherryRNAi*) stained with anti-BiP. A second UAS line does not influence BiP induction in frataxin-deficient flies. **(B)** Representative image of 35-day-old frataxin-deficient GGCs coexpressing a *MarfRNAi* construct (*fhRNAi-1*/+; Repo-GAL4/ UAS-*MarfRNAi*) stained with anti-BiP. Clear suppression of BiP induction by *Marf* silencing. **(C)** Representative image of 35-day-old control GGCs (*Repo*-GAL4/ UAS-*MarfRNAi*) stained with anti-BiP. In contrast to muscles (Debattisti et al., 2014), *Marf* downregulation in glia did not induce BiP expression. **(D)** Representative image of 35-day-old frataxin-deficient GGCs coexpressing *Marf* downregulation and the Xbp1GFP reporter (*fhRNAi-1*/UAS-*Xbp1GFP*; *Repo*-GAL4fUAS-*MarfRNAi*) stained with anti-BiP. Nuclear localization of Xbp1 and BiP expression were restored to wild type levels. **(E)** A second RNAi line to downregulate Marf expression also reduces BiP immunoreactivity in FRDA flies (*fhRNAi-1*/+; *Repo*-GAL4/UAS-*MarfRNAi*(TRiP)). In all cases, scale bars represent 40 μm.

Next, we addressed whether *Marf* was modulating the ER stress response in frataxin-deficient glia. Remarkably, *Marf* downregulation using two independent RNAi lines was sufficient to strongly reduce BiP expression in 80% of the frataxin-deficient flies analyzed (Figures 8B,D,E) compared to frataxin-deficient flies coexpressing mcherryRNAi (Figure 8A) and to restore expression of spliced Xbp1 in frataxin-deficient GGCs (Figure 8D). It is important to note that *Marf* downregulation in glia did not seem to trigger any ER stress response by itself (Figure 8C), whereas it does in *Drosophila* muscles (Debattisti et al., 2014). Our results suggest that mitofusin mediates the ER stress response in frataxin-deficient glia and therefore *Marf* downregulation is able to compensate the altered ER stress response and concomitantly reverses the locomotor dysfunction, the brain degeneration and the accumulation of lipid droplets (Figures 1A,C,I), the main hallmarks of frataxin downregulation in the *Drosophila* glia (Navarro et al., 2010).

**Figure 9:**
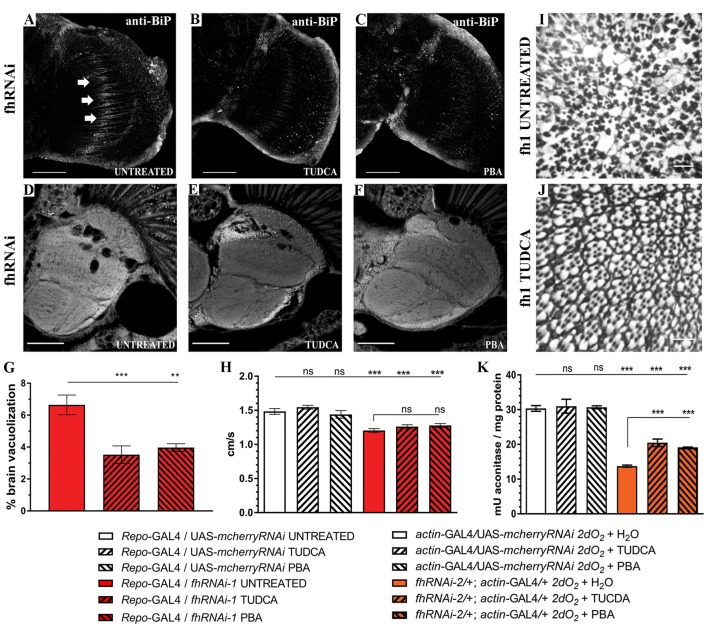
Chemical reduction of ER stress improves FRDA conditions. **(A–C)** Representative images of 35-day-old frataxin-deficient (Repo-GAL4/*fhRNAi-1*) GGCs stained with anti-BiP to monitor ER stress levels. Clear reductiion of BiP induction by tauroursodeoxycholic acid (TUDCA) **(B)** and 4-phenylbutyric acid (4-PBA) **(C)** treatments compared to untreated flies **(A)**. **(D–F)** Representative images of paraffin brain sections from 35-day-old flies displaying frataxin knockdown in glial cells (*fhRNAi-1*; *Repo*-GAL4) untreated **(D)** or treated with 15 mM TUDCA **(E)** and 7.5 mM PBA **(F)**. **(G)** Quantification of degenerated area (%) in 35-day-old frataxin-deficient flies. Treatment with chemical chaperones such as TUDCA and PBA partially rescued brain vacuolization in FRDA flies. **(H)** Negative locomotor performance of 5-day-old controls and FRDA flies treated with TUDCA and PBA. No recovery of locomotor deficit in frataxin-deficient flies. **(I,J)** Representative images of retinas of 5-day-old fh1 mutant clones either untreated **(I)** or treated with TUDCA **(J)**. TUDCA administration seems to prevent photoreceptor neuron degeneration. **(K)** TUDCA and PBA supplementation protect aconitase activity. Frataxin-deficient flies showed a strong reduction of the enzymatic activity under oxidative insult. Administration of TUDCA and PBA increased aconitase activity by 25%. Scale bars represent 40 μm in **(A–C)**, 50 μm in **(D–F)** and in 10 μm **(I,J)**. In all graphs data is shown as means ± SEM. Data was analyzed by One-way ANOVA with *post hoc* Tukey Multiple Comparison Test. **P < 0.01; ***P < 0.001.

Finally, to further analyze the impact of ER stress in our FRDA model, we decided to test whether two chemical chaperones, TUDCA and 4-PBA, which have been shown to attenuate ER stress (Debattisti et al., 2014; Celardo et al., 2016), were sufficient to improve frataxin-deficient phenotypes. Uptake of 15 mM TUDCA or 7.5 mM PBA was able to clearly reduce the BiP immunoreactivity of FRDA flies (Figures 9A–C) in the region of interest (arrows in Figure 9A) and concomitantly, both were able to promote a strong reduction of around 45% of the brain vacuolization triggered by frataxin depletion in glia (Figures 9D–G) which is similar to *Marf* downregulation (65%, Figure 1F). However, TUDCA and PBA did not improve the locomotion defects induced by glial-targeted frataxin silencing (Figure 9H) suggesting that other factors besides ER stress might be involved in this defect. Following the strategy described by Chen et al. (2016b), we generated mosaic mutant clones of *Drosophila* photoreceptor neurons by means of the Flippase/Flippase Recognition Target (FLP/FRT)-system and we analyzed the morphology of the photoreceptors in 5-day-old flies upon treatment with TUDCA. Compared to untreated flies (Figure 9I), application of the drug during development and adulthood helped to maintain the structural integrity of the photoreceptors and reduced the degeneration (Figure 9J). We and others have already shown that frataxin silencing with *fhRNAi-2* decreases aconitase activity under oxidative stress (Llorens et al., 2007; Navarro et al., 2010, 2015). Moreover, this model has been reported as an interesting tool to test drugs that might improve FRDA conditions (Soriano et al., 2013; Calap-Quintana et al., 2015). As shown in Figure 9K, TUDCA and PBA were able to moderately increase aconitase activity (25%) in frataxin deficient flies (*fhRNAi-2*/+; *actin*-GAL4/+) without affecting the aconitase activity of controls (*actin*-GAL4/UAS-*mcherryRNAi*).

These experiments clearly indicate a positive effect by reducing the ER stress in frataxin-deficient flies and links ER stress with the neurodegeneration observed in the FRDA nervous system. Our results highlight ER stress as a crucial mediator in FRDA as well as a new cellular process for therapeutic interventions.

## Discussion

FRDA is the most common recessive ataxia in the Caucasian population. Reduction of frataxin expression affects the synthesis of iron-sulfur clusters as well as the cellular metabolism of iron and the mitochondrial production of ATP leading to mitochondrial dysfunction and concomitant cellular degeneration and death. In the last decade, *Drosophila* has become one of the most powerful and attractive models to study targeted frataxin deficiency in several tissues such as peripheral and central nervous system, glia and heart (Anderson et al., 2008; Navarro et al., 2010; Shidara and Hollenbeck, 2010; Tricoire et al., 2014; Chen et al., 2016b) as well as in the whole organism (Anderson et al., 2005; Llorens et al., 2007; Calap-Quintana et al., 2015).

FRDA is a mitochondrial pathology and indeed, alterations in mitochondrial morphology ranging from enlarged organelles (Bolinches-Amorós et al., 2014; Obis et al., 2014) to increased fragmentation (Lefevre et al., 2012; Schiavi et al., 2015) have been reported in FRDA models. However, very few reports have carried out a systematic and comprehensive analysis of the mitochondrial dynamics in cells lacking frataxin. We have addressed this question using our model for frataxin-defiency in glial cells (Navarro et al., 2010, 2015). We initially performed a forward genetic screen to decipher more genetic interactions linking mitochondrial dynamics and frataxin knockdown. Our results revealed the *Drosophila Mitofusin* homolog (*Marf*) as a key mediator of the pathology in the glia. *Marf* downregulation was able to fully recuperate some of the most important phenotypes induced by frataxin silencing in glia such as the locomotor dysfunction, the brain degeneration and the accumulation of lipid droplets in the brain (Navarro et al., 2010). In agreement with the reduction of lipid accumulation observed in this work upon *Marf* knockdown, *Marf* mutants also display reduced number of lipids (Sandoval et al., 2014). Importantly, *Marf* silencing did not affect frataxin levels (Supplementary Figure S6A).

The strong genetic interaction with *Marf* initially suggested critical alterations of mitochondrial dynamics in frataxin-deficient cells. Studies in *Drosophila* muscles have provided remarkable evidence regarding mitochondrial homeostasis (Clark et al., 2006; Deng et al., 2008; Wang et al., 2016). Because of this, we decided to establish *Drosophila* IFM as a platform to study frataxin deficiency. Frataxin downregulation in this mitochondrial-enriched tissue recapitulates the basic physiological and biochemical defects observed in patients (Beauchamp et al., 1995; Lynch et al., 2002; Nachbauer et al., 2012). Frataxin deficiency in *Drosophila* muscles induced mitochondrial fragmentation that was accompanied by changes in the expression levels of genes involved in mitochondrial fusion and fission (*Opa1, Marf and Drp1*) and in mitochondrial quality control (*Pink1*). However, a similar analysis performed in yeast did not report any difference (Lefevre et al., 2012). Interestingly, three of these modifications (upregulation of *Opa1* and downregulation of *Drp1* and *Pink1*) are consistent with the promotion of mitochondrial fusion. However, since fragmentation is critical to avoid “contamination” of healthy mitochondria through fusion with damaged or senescent mitochondria (Bhandari et al., 2014), the observed expression profile might represent a pathological downstream consequence of frataxin deficiency.

Mitochondrial fragmentation and perinuclear clustering normally precedes clearance by means of a special form of autophagy called mitophagy (Okatsu et al., 2010; Palikaras and Tavernarakis, 2014). We have detected in glia and muscles of FRDA flies that damaged mitochondria are correctly labeled and targeted for degradation, indicating an effective integration of mitochondrial checkpoints. However, we also detected accumulation of the autophagy marker p62. A similar result has also been found in the cardiac tissue and sensory neurons of an FRDA mouse model (Huang et al., 2013; Mollá et al., 2017, respectively). Moreover, lysosomes and autophagosomes seem to accumulate in the DRG of FRDA rodent models (Simon et al., 2004; Al-Mahdawi et al., 2006). Several reports have linked such accumulations to a deficient autophagy process (reviewed in Mauvezin et al., 2014). Thus, it is necessary to elucidate whether frataxin silencing induces or blocks autophagy. This question has recently been addressed in *C. elegans* (Schiavi et al., 2015). Using histological markers, the authors concluded that the autophagic flux remained intact. However, the worm possesses the peculiarity of being the only FRDA model that displays an extended longevity upon strong frataxin depletion. Therefore, we decided to perform a detailed investigation of the autophagy-mitophagy process in two different tissues, muscle and glia, in a *Drosophila* FRDA model that shows the expected shortened lifespan (this work and Navarro et al., 2010).

In our approach, we have shown that autophagosomes are properly recruited and autophagolysosomes are also generated. In addition, our molecular analysis of the turnover rate of the autophagy markers suggested an enhanced flux which was much more evident in glia than in muscles. The careful analysis of the mtRosella marker clearly showed that the majority of damaged organelles have lost both, the pH-sensitive and the pH-insensitive markers, as a strong indication of functional mitophagy. The mtRosella transgene has already been demonstrated to be a powerful tool to analyze mitophagy *in vivo* in yeast (Rosado et al., 2008; Böckler and Westermann, 2014), worms (Schiavi et al., 2015) and mammalian systems (Sargsyan et al., 2015). Our generation of the first fly stock that expresses this construct incorporates *Drosophila* to this group of model organisms and further expands the *Drosophila* tools to study autophagy and mitophagy. One of the main problems in the field of frataxin research is the lack of homogeneous results between different models. The information given by a specific readout or marker might reflect the singularity of each model or each experimental scenario. This work highlights that induction of mitophagy is a physiological hallmark in each single FRDA model and it might represent a new pathway to unveil potential biomarkers of the disease.

Interestingly, our analysis has also revealed that expression of a GFPATG8a marker was sufficient to reduce p62 accumulation as well as to improve brain degeneration. This result might suggest that on the one hand, GFPATG8a is not a neutral marker and it is potentially generating a gain-of-function situation that stimulates autophagy and that on the other hand, induction of autophagy might also be a beneficial therapeutic approach in FRDA. Although there is still controversy about the functionality of ATG8a markers, their ability to work as wild type proteins has also been shown for other ATG8a constructs (Pircs et al., 2012). Our result was further supported by the effects observed upon overexpression of endogenous ATG8a by means of an independent UAS-ATG8a line. Unfortunately, the hypothesis could not be tested by overexpression of another key autophagy gene such as ATG1 since its expression resulted in lethality when expressed in glia. On the contrary, expression of LAMP1GFP did not exert any protective function, likely because it consists of the fusion between specific domains of human LAMP1 and eGFP (Jacomin et al., 2016) and this might explain the lack of an effect. The positive influence of autophagy suggests that specific enhancing of mitophagy might have similar effects. Parkin overexpression seemed an attractive possibility since Parkin promotes mitophagy in mammalian cells (Geisler et al., 2010). In addition, Marf is ubiquitinated by Parkin (Ziviani et al., 2010) and thus, increasing Parkin expression would also reduce the amount of Marf. However and in contrast to ATG8a, overexpression of *Parkin* failed to modify the brain degeneration or p62 accumulation in our experimental scenario. This is not completely surprsing since several susbstrates have been predicted to be targets of Parkin in *Drosophila* (Martinez et al., 2017) and thus, *Parkin* overexpression and *Marf* silencing represent two complete different situations. On the other hand, *Marf* silencing seems to improve Parkinson’s disease phenotypes independently of the autophagy process (Liu and Lu, 2010; Celardo et al., 2016). *Marf* knockdown has been shown to completely suppress *Pink1* and *Parkin* loss of function in *Drosophila* models of Parkinson’s disease in muscles (Deng et al., 2008; Liu and Lu, 2010) and in the heart (Bhandari et al., 2014) by preventing mitochondrial fusion. Interestingly, another report has recently demonstrated that in the nervous system of two fly models of Parkinson’s disease, *Marf* knockdown rescued phenotypes influencing ER stress (Celardo et al., 2016) and not mitochondrial dynamics. Remarkably, human and fly mitofusins have been already described to modulate cellular responses to ER stress (Muñoz et al., 2013; Debattisti et al., 2014). All these evidences along with the lack of rescue by *Opa1* downregulation, *Drp1* overexpression (both theoretically equivalent to *Marf* knockdown) and *Parkin* overexpression in our FRDA model suggested that *Marf* should be influencing another process in frataxin-deficient flies.

Increased activity and expression of ER stress markers such as the activating transcription factor 4 (ATF4), the chaperone binding immunoglobulin protein (BiP) and phosphorylated eIF2α have been found in cellular (Cortopassi et al., 2006; Lu and Cortopassi, 2007) and mouse (Huang et al., 2013) FRDA models. In other cases, frataxin depletion sensitized cells to chemically-induced ER stress and triggered accumulation of the chaperone BiP/Grp78 (Cnop et al., 2012; Bolinches-Amorós et al., 2014). Interestingly, ER stress markers are induced even before some Fe-S dependent enzymatic activities started to decline in FRDA (Lu and Cortopassi, 2007). The activation of ER stress responses in FRDA is consistent with different hypotheses, such as aggregation of misfolded Fe-S proteins, redox alterations and abnormal calcium metabolism. The pathogenic role of ER stress has been already highlighted in other *Drosophila* models of neurodegenerative diseases (Celardo et al., 2016; Sanchez-Martinez et al., 2016; López Del Amo et al., 2017). In agreement, we have observed in our models an altered ER stress response in glia and muscles. We could detect changes in all three branches of the UPR by means of increased expression of BiP, abnormal induction of spliced Xbp1 and enhanced phosphorylation levels of eIF2α. Unexpectedly, we found strong induction of the spliced form of Xbp1 and its translocation to the nucleus in glia of control flies, whereas this response was completely abrogated in FRDA flies. The current studies addressing the ER stress response in glial cells are limited. Constitutive activation of the IRE/Xbp1 pathway has already been described in larval glial cells and it seems to be related to their role in generating all the components for the multi-layered membrane sheaths around neurons (Sone et al., 2013). Interestingly, the GGCs seem to belong to the subpopulation of ensheathing glia in the adult brain (Kremer et al., 2017). Therefore, impaired Xbp1 activation might affect axonal ensheathment and concomitantly certain protective roles of glial cells leading to the brain degeneration observed in these flies. Moreover, loss of function of *Drosophila* MANF, a protein produced in glial cells, also reduces the expression of spliced Xbp1 (Lindström et al., 2016). Besides glia, other tissues such as larval intestine, fat body, certain neurons, developing photoreceptors, male reproductive system and the adult intestinal stem cells also show default activation of XBP1-based reporters (Ryoo, 2015). Such activation seems to be pivotal for a proper tissue homeostasis.

Notably, our results demonstrate that *Marf* knockdown suppresses the ER stress induced by frataxin-deficiency. Therefore, our results indicate that this might be the most important mechanism underpinning the rescue driven by *Marf* silencing in FRDA glial cells. This hypothesis has been further corroborated by feeding the flies with a couple of chemical chaperones, TUDCA and PBA, which counteract ER stress including lipid-dependent ER stress (Pineau et al., 2009; Liu et al., 2014). Remarkably, chemical reduction of ER stress was sufficient to improve brain integrity and photoreceptor degeneration. We have also found that both compounds were able to partially suppress aconitase inactivation in FRDA flies. These results highlight ER stress as a very attractive and promising therapeutic approach to treat FRDA. It has been reported that TUDCA and PBA might also act as antioxidant agents (Liu et al., 2002; Oveson et al., 2011). Moreover, antioxidants have been suggested as potential beneficial treatments in FRDA patients and mouse models (Seznec et al., 2004; Parkinson et al., 2013, respectively). Our experiments coexpressing three different antioxidant enzymes showed that, in agreement with Anderson et al. (2008), only hydrogen peroxide scavengers (mitochondrial catalase, *mitocatalase* and mitochondrial thioredoxin reductase, UAS-*TrxmitoB*) seemed to display some positive influence on fly locomotion. However, both failed to improve brain vacuolization and the presence of lipid droplets (Supplementary Figure S7). These results, along with those reported by other *Drosophila* FRDA models (Tricoire et al., 2014; Chen et al., 2016b), strongly question antioxidants as effective therapeutic agents in FRDA and highlight the relevance of protection mediated by *Marf* silencing. The lack of effects upon rapamycin treatment is also in agreement with this idea since it has been suggested to activate antioxidant pathways in a fly model of FRDA (Calap-Quintana et al., 2015) in contrast to yeast in which rapamycin reduced mitochondrial mass likely by stimulating mitophagy (Marobbio et al., 2012).

We have also found differences in relation to the severity of the defects and the nonsynchronous progression of the pathology in different tissues. We speculate that glial mitochondria are progressively targeted for degradation whereas in muscles the accumulation of damaged organelles is too substantial in a very short period of time. The mitochondria labeled for clearance cannot be countered at the same speed by the mitophagic machinery and this, and not an impairment of mitophagy, triggered the accumulation of p62. In addition, the impact of other genes in the biology of glia and muscles might also explain the lack of consistency between our genetic screenings. For example, *Marf* downregulation affects locomotion in muscles (Supplementary Figure S6B) but not in glia (Figure 1A) and induces ER stress in muscles (Debattisti et al., 2014) but not in glia (this work).

**Figure 10:**
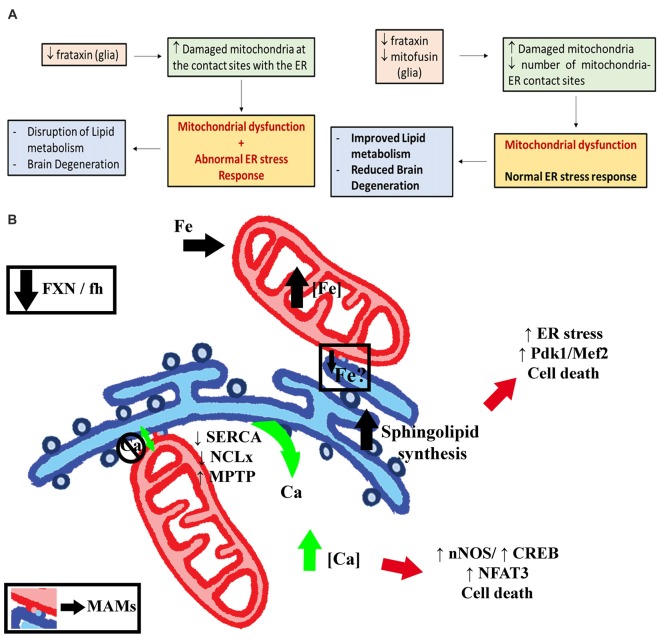
Proposed model for *Marf*-mediated ER stress in FRDA flies. **(A)** Model suggested by our results. In short, mitofusin regulates the ER stress response in frataxin-deficient glia likely by mitochondria-ER tethering. *Marf* silencing is able to normalize the altered ER stress response and concomitantly to reverse the locomotor dysfunction, the brain degeneration and the accumulation of lipid droplets. **(B)** Hypothetical graphical model showing that ER-mitochondrial contacts might be mediating different phenotypes described in FRDA models. On the one hand, frataxin deficiency increases mitochondrial iron concentration. In parallel, iron stimulates sphingolipid synthesis in the ER. Sphingolipids activate the Pdk1/Mef2 pathway and might induce ER stress leading to cell death. Whether a transport of iron from the mitochondria to the ER is underpinning this effect still has to be demonstrated (Fe?). On the other hand, frataxin downregulation in mammalian cells has been shown to abolish mitochondrial buffering capacity for calcium. This results in an increased concentration of cytosolic calcium by inhibiting SERCA pumps, downregulating the calcium transporter NCLx and opening the MPTP. High calcium activates the nNOS pathway and the transcription factors CREB and NFAT3, leading also to cell death. Importantly, some crucial sphingolipids are synthesized in the MAMs and MAMs play critical roles in the regulation of Calcium homeostasis. Ca, Calcium; CREB, cAMP response element-binding protein; ER, Endoplasmic Reticulum; Fe, Iron; MAMs, Mitochondria-associated membranes, Mef2, myocyte enhancer factor-2; MPTP, Mitochondrial Permeability Transition Pore; nNOS, Neuronal nitric oxide synthases; Pdk1, 3-phosphoinositide dependent protein kinase-1.

Mitofusins were some of the first proteins shown to be present at the core of the interface between mitochondria and ER, namely mitochondria-associated membranes (MAMs; de Brito and Scorrano, 2008). MAMs are crucial mediators of several processes such as calcium signaling, apoptosis, autophagy and lipid biosynthesis (reviewed in Krols et al., 2016b; Paillusson et al., 2016). In addition, MAMs have already been linked to other neurodegenerative disorders, such as Alzheimer’s disease and amyotrophic lateral sclerosis (Schon and Area-Gomez, 2013; Manfredi and Kawamata, 2016). Due to methodological limitations, subcellular compartments cannot be distinguished in *Drosophila* glial cells in electron microscope preparations and thus, we could not quantify the number of MAMs in controls and in FRDA glial cells. Importantly, loss of *Marf* has already been shown to reduce the contacts between mitochondria and ER in the ring gland (Sandoval et al., 2014) and in fly neurons (Celardo et al., 2016). Remarkably, reduction of mitochondria-ER contacts was sufficient to reduce ER stress and rescue phenotypes in two *Drosophila* models of Parkinson’s disease (Celardo et al., 2016). All these facts along with the improvement of ER stress upon *Marf* silencing and the positive effect of chemical chaperones on FRDA phenotypes suggest that the role of *Marf* in the mitochondria-ER axis might be a key mechanistic element in our fly models. Our results are also important in light of the mechanisms recently described in a new fly model of FRDA (Chen et al., 2016b). Chen and collaborators found that iron mediates activation of the sphingolipid metabolism that in turn induces neurodegeneration in fly photoreceptors via the Pdk1/Mef2 pathway. Interestingly, the same pathway is active in the nervous system of frataxin-deficient mice as well as in heart samples from FRDA patients (Chen et al., 2016a). Importantly, sphingolipids are synthesized in the ER in mammals (Filadi et al., 2017) and flies (Hebbar et al., 2015) and increased levels have been shown to elicit the ER stress response in mice (Contreras et al., 2014), cells (Fu et al., 2011; Liu et al., 2014), flies (Hebbar et al., 2015) and yeast (Pineau et al., 2009). Furthermore, some sphingolipids such as ceramide (which is increased in flies and mouse FRDA models as well as in patients) are specifically synthesized in MAMs (Stiban et al., 2008; Hayashi and Fujimoto, 2010). Moreover, we already described a few years ago that frataxin silencing in glia induced accumulation of lipids (Navarro et al., 2010) and that enhanced iron import into the mitochondria was important in the FRDA glial phenotypes (Navarro et al., 2015). Therefore, it is appealing to speculate that the iron surplus in FRDA might travel from mitochondria to the ER through the MAMs to stimulate the iron-dependent sphingolipid production. However, such a mechanism has not yet been resolved. Alterations of calcium homeostasis are also known to induce ER stress (Pineau et al., 2009; Fu et al., 2011; Contreras et al., 2014; Liu et al., 2014). Regulation of calcium metabolism is another role that MAMs play in the cell (Krols et al., 2016a). Interestingly, calcium dyshomeostasis has been found in different cellular models lacking frataxin (Bolinches-Amorós et al., 2014; Mincheva-Tasheva et al., 2014; Mollá et al., 2017; Purroy et al., 2018). In these cases, frataxin silencing seems to deplete ER calcium stores by inhibiting the SERCA pump (Bolinches-Amorós et al., 2014). Simultaneously, the expression of the calcium transporters NCL_x_ and the mitochondrial permeability transition pore (MPTP) is also altered (Purroy et al., 2018). All this will increase cytosolic calcium concentration, that will become toxic in the absence of the buffering capacity of the mitochondria and will activate neuronal nitric oxide synthase (nNOS), the cAMP response element-binding protein (CREB) pathway (Mincheva-Tasheva et al., 2014) and the NFAT3 factor (Purroy et al., 2018) leading to cellular hypertrophy and cell death. Unfortunately, this aspect has never been addressed in the fly FRDA models. All these evidences from the literature in combination with the results presented in this work (Figure 10A) might allow developing a unifying hypothetical model (Figure 10B) in which reduction of frataxin expression impairs iron-sulfur cluster production and mitochondrial function. This, in turn, increases the number of defective mitochondria at the ER contacts and disturbs mitochondrial iron, lipid and calcium homeostasis. All these defects boost ER stress signaling through MAMs leading to cellular degeneration and cell death.

## Conclusion

We have demonstrated that *Marf* downregulation rescues the locomotion and the accumulation of lipid droplets triggered by targeted frataxin depletion in glia along with nervous system degeneration. The analysis of the mechanism underlying such recovery has allowed us to underscore that alterations in the ER biology are pivotal in the neurodegeneration in FRDA. Our results suggest that *Marf* and ER stress represent a hub in the neurodegenerative process of FRDA and uncover both as important elements substantially involved the FRDA pathology.

## Availability of Data and Materials

All data generated and analyzed during this study are included within the article and its additional files. The raw data obtained and/or analyzed during the current study are available from the corresponding author on reasonable request. Data sharing is not applicable to this article as no datasets were generated or analyzed during the current study.

## Ethics Statement

This manuscript involves the use of *Drosophila melanogaster*. It is important to note down that the European Directive 2010/63/EU on the protection of animals used for scientific purposes does not apply to *Drosophila melanogaster* and therefore *Drosophila* procedures and protocols do not need to be approved by Research Ethical Committees.

## Author Contributions

OE and JAN designed the study, conducted experiments, and analyzed data. OE, SS and JAN wrote the manuscript. All authors have been involved in drafting the manuscript and revising it critically for important intellectual content. All authors read and approved the final manuscript.

## Conflict of Interest Statement

The authors declare that the research was conducted in the absence of any commercial or financial relationships that could be construed as a potential conflict of interest.

## Footnotes

^1^www.graphpad.com

## Supplementary Material

FIGURE S1 Behavioral and mitochondrial defects triggered by frataxin downregulation in fly muscles. **(A)** Two independent RNAi lines induce a frataxin downregulation in thoracic muscles using *Mef2*-GAL4 from 60% (*fhRNAi-2*) to 90% (*fhRNAi-1*). **(B)** Targeted frataxin knockdown in muscles was sufficient to shorten mean and maximum life span. **(C)** Frataxin silencing in muscles impairs negative geotaxis already at 3 days after eclosion and completely abolished locomotion after 5 days upon severe downregulation and after 15 days upon moderate silencing. **(D)** Frataxin depletion in the fly musculature induces hypersensitivity towards oxidative insult. **(E)** Aconitase activity is clearly diminished in frataxin-deficient muscles 5 days post eclosion. **(F)** ATP production is severely affected in frataxin-deficient flies (50%) and the age-dependent decay of ATP is enhanced upon frataxin-silencing. **(G–J)** In order to analyze individual differences between organelles, we stained the mitochondria with the permeable dye tetramethylrhodamine, ethyl ester (TMRE), which only accumulates inside of mitochondria, which are active and which have a normal membrane potential. **(G)** TMRE staining of 1-day-old control muscles. Perfect colocalization between mitoGFP and TMRE signals indicating functional mitochondria. **(H)** TMRE staining of 1-day-old Friedreich’s ataxia (FRDA) muscles. Arrows indicate lack of colocalization between mitoGFP and TMRE signals in some organelles suggesting impaired mitochondrial membrane potential already in young flies. **(I)** TMRE staining of 7-day-old control muscles. Perfect colocalization between mitoGFP and TMRE signals. **(J)** TMRE staining of 7-day-old FRDA muscles. No colocalization between mitoGFP and TMRE signals in most mitochondria indicating that membrane potential is abolished. **(K)** Magnification from **(J)**. TMRE staining of 7-day-old FRDA muscles. Arrows denote representative mitochondria in which TMRE is not incorporated into the organelle due to a deficient membrane potential indicating severe mitochondrial dysfunction. This is consistent with the biochemical dysfunctions described in **(E,F)**. **(L)** Quantification of mitochondrial TMRE signal in controls and frataxin deficient mitochondria. Clear reduction of mitochondrial to cytoplasmic ratio in 1-day-old FRDA flies suggesting a mitochondrial dysfunction already at this age. Age worsened the phenotype. Scale bars represents 3 μm in **(K)** and 10 μm in the rest of panels. Graphs represent means ± SEM. Data were analyzed by Unpaired *T*-test in **(E,F)**, by Log-rank (Mantel-Cox) and Gehan-Breslow-Wilcoxon tests in **(B,D)** and by one-way ANOVA with *post hoc* Tukey Multiple Comparison test in **(A,C,L)**. **P* < 0.05; ***P* < 0.01; ****P* < 0.001.

FIGURE S2 Analysis of age-dependent accumulation of p62-positive vesicles upon frataxin silencing in fly muscles. **(A)** MitoGFP labeled mitochondria from 1-day-old controls (UAS-*mitoGFP*/+; *Mef2*-GAL4/UAS-*mcherryRNAi*). **(B)** MitoGFP labeled mitochondria from 1-day-old frataxin-deficient thoraces (UAS-*mitoGFP*/+; *Mef2*-GAL4/*fhRNAi-1*). **(C)** MitoGFP labeled mitochondria from 25-day-old controls (UAS-*mitoGFP*/+; *Mef2*-GAL4/UAS-*mcherryRNAi*). **(D)** MitoGFP labeled mitochondria from 25-day-old frataxin-deficient thoraces (UAS-*mitoGFP*/*fhRNAi-2*; *Mef2*-GAL4/*+*) A second RNAi also induces some mitochondrial fragmentation but only in old flies. **(E–G)** Relative expression levels of genes involved in mitochondrial quality control and dynamics. Our results showed that *Opa1* (involved in fusion) was upregulated and dynamin-related protein 1 (*Drp1*) (involved in fission) was clearly downregulated. Surprisingly, and in contrast to *Opa1*, *Drosophila Mitofusin* (*Marf*), a second gene involved in mitochondrial fusion, was slightly downregulated. In muscles, *Marf* also seems to behave differently to its counterparts in fusion and fission of mitochondria. *Pink1* expression was reduced around 50% whereas the expression of *parkin*, Pink1’s partner in the activation of mitophagy, was not affected. We also found that the transcriptional coactivator *spargel* (*Srl*) and the transcription factor *tfam*, both involved in the biosynthesis of mitochondria, were not altered in the FRDA muscle cells. Similar results were obtained in old *fhRNAi-2* flies. **(H–H″)** Representative pictures of 1600 μm^2^ from 1 **(H)**, 5 **(H′)** and 10 **(H″)** day-old frataxin-deficient muscles stained with anti-P62 and used to calculate the results represented in **(I,J)**. **(I)** Analysis of size of p62 vesicles in frataxin-deficient flies at three different time points (1, 5 and 10-day-old flies). We observed a reduction around 30% after 5 days and 50% after 10 days. This result is consistent with the increased mitochondrial fragmentation reported in Figure 3B. **(J)** Quantification of p62 vesicles in frataxin-deficient flies at 3 different ages (1, 5, 10 days). The number of vesicles increased by 8-fold at 10 days compared to 1 day. **(K)** mRNA expression levels of *Drosophila p62* in control and frataxin-deficient muscles. *p62* expression is only slightly upregulated in FRDA flies. **(L)** Magnification from Figure 3D. p62 signals form vesicular structures that engulf mitochondria as part of the *in vivo* clearance process. **(M)** Quantification of mtDNA content in frataxin-deficient muscles by semiquantitative Real Time PCR. Clear accumulation of mtDNA in FRDA flies. The method was validated reproducing the reported content in the *Pink1B9* mutant allele. **(N–P)** Representative confocal pictures from controls (**N**, UAS-*mitoGFP*/+; *Mef2*-GAL4/UAS-*mcherryRNAi*), parkin-deficient muscles (**O**, UAS-*mitoGFP*/+; *Mef2*-GAL4/UAS-*parkinRNAi*) and pink1-deficient muscles (**P**, UAS-*mitoGFP*/+; *Mef2*-GAL4/UAS-*Pink1RNAi*). Mitochondria were labeled using mitoGFP and mitophagy was monitored with anti-p62. Defects in mitochondrial morphology could be detected upon *parkin* and *pink1* silencing **(O,P)**. Interestingly, p62 staining seemed to be enhanced in *parkin* and *pink1* deficient muscles but p62 was not recruited to the enlarged mitochondria (arrows in **O,P**). This corroborates that functional *parkin* or *pink1* are required to recruit p62 into defective mitochondria. **(Q–T)** Representative confocal pictures from controls (**Q,S**, UAS-*mitoGFP*/+; *Mef2*-GAL4/UAS-*mcherryRNAi*) and frataxin deficient flies using the second RNAi line (**R,T**, UAS-*mitoGFP*/*fhRNAi-2*; *Mef2*-GAL4/*+*). Accumulation of p62 was only noticeable in old FRDA flies **(T)**. Scale bars represent 10 μm in all except for **(L)** that represents 1 μm. All graphs represent means ± SEM. Data was analyzed by Unpaired *T*-test in **(E,F,G,K,M)** and by one-way ANOVA with *post hoc* Dunnett Multiple Comparison test in **(I,J)**. **P* < 0.05; ***P* < 0.01; ****P* < 0.001.

FIGURE S3 Histological analysis of autophagy markers in control flies. **(A)** Representative LAMP1GFP and p62 patterns in the GGCs of a 35-day-old control fly (UAS-*LAMP1GFP*/+; *Repo*-GAL4/UAS-*mcherryRNAi*). Controls presented little amounts of LAMP1 and p62 structures, indicative of basal autophagic levels. **(B)** Representative LAMP1GFP and p62 patterns in the GGCs of a 35-day-old FRDA fly (UAS-*LAMP1GFP*/+; *Repo*-GAL4/*fhRNAi-1*). Increased p62 signals. LAMP1GFP immunoreactivity is enhanced nearby the p62 vesicles suggesting the formation of autophagolysosomes to degrade mitochondria. Arrows denote colocalization of LAMP1 and p62 signals. Unfortunately, the reduced size of organelles in glial cells hampered an accurate analysis. **(C)** Representative GFPATG8a and p62 patterns in GGCs of a 35-day-old control fly (*Repo*-GAL4/ UAS-*GFPATG8a*). **(D)** In glia, besides some cytoplasmisc localitation, GFPATG8a also displayed a clear nuclear pattern as it colocalized with the glial-specific transcription factor Repo (arrows). This nuclear distribution is in agreement with the findings reported for fly GFPATG8a and mammalian LC3/ATG8 (Mauvezin et al., 2014; Huang et al., 2015), respectively). This localization is necessary for the deacetylation of LC3 by Sirt1. Loss of acetyl groups at two specific lysine residues is required for interaction with ATG7 that triggers the formation of autophagosomes (Huang et al., 2015). **(E)** Quantification of GFPATG8a and LAMPGFP western blots from muscles. Clear increase of Free GFP upon frataxin silencing also in muscles. This result might suggest increased mitophagic flux in muscles too. **(F)** Representative picture of LAMP1GFP and p62 patterns in 5-day-old control muscles (UAS-*LAMP1GFP*/+; *Mef2*-GAL4/UAS-*mcherryRNAi*). **(G)** Representative picture of GFPATG8a and p62 patterns in 5-day-old control flies (*Mef2*-GAL4/UAS-*GFPATG8a*). Scale bars represent 40 μm in **(A–D)** and 10 μm in **(F,G)**. Graph in **(E)** represents means ± SEM. Statistical analysis was performed by Unpaired *T*-test. **P* < 0.0; ****P* < 0.001.

FIGURE S4 Effects of genetic and chemical induction of autophagy in frataxin-deficient flies. **(A)** Representative GFPATG8a and p62 patterns in the optic lobe of a 35-day-old FRDA fly (*fhRNAi-*1/+; Repo-GAL4/UAS-*GFPATG8a*). Interestingly, no p62 accumulation is detected in the GGCs region. Arrows denote autophagosomes. **(B)** Representative semithin epon plastic section of a 35-day-old frataxin-deficient fly coexpressing GFPATG8a. Brain vacuolization in the lamina and in the outer chiasm was significantly reduced. However, GFPATG8a coexpression was not sufficient to counteract the accumulation of lipid droplets (arrows), another hallmark of frataxin-deficiency in *Drosophila* glia (Navarro et al., 2010). **(C)** Representative semithin epon plastic section of a 35-day-old frataxin-deficient fly coexpressing LAMP1GFP. A strong vacuolization (asterisks) is detected in the lamina and in the outer chiasm. The presence of lipid accumulation is denoted by droplets distributed throughout the brain (arrows as example). **(D)** Quantification of degenerated area (%) in 35-day-old frataxin-deficient flies expressing UAS-*LAMP1GFP*, UAS-*GFPATG8a* and UAS-*ATG8a* compared to coexpression of UAS-*GFP* analyzed area. Vacuolization was improved upon expression of ATG8a transgenes. **(E)** Representative paraffin brain section from a 35-day-old fly displaying frataxin knockdown and expression of ATG8a in glial cells (*fhRNAi-1*/+; *Repo*-GAL4/UAS-*ATG8a*). Strong reduction of brain vacuolization was observed. **(F)** Representative p62 patterns in the optic lobe of a 35-day-old FRDA fly coexpressing UAS-*ATG8a*. No p62 accumulation was detected in the GGCs region. **(G)** p62 levels in frataxin-deficient muscles overexpressing autophagy markers GFPATG8a and LAMP1GFP. Muscles with increased expression of GFPATG8a showed lower levels of p62 compared to individuals overexpressing either the lysosomal marker LAMP1GFP or an unrelated marker (ERGFP). **(H)** Negative locomotor performance of 5-day-old controls and flies displaying glial-specific frataxin silencing treated with rapamycin. **(I)** Quantification of degenerated area (%) in FRDA flies treated with rapamycin or with control food (DMSO). Rapamycin was unable to improve the locomotor deficit and the brain degeneration present in frataxin-deficient flies. Scale bars represent 50 μm in **(B,C,E)** and 40 μm in **(A,F)**. All graphs are represented as means ± SEM. Statistical analysis was performed by one-way ANOVA with *post hoc* Dunnett Multiple Comparison test in **(D,G,H)** and Unpaired *T*-test in **(I)**. ***P* < 0.01; ****P* < 0.001.

FIGURE S5 ER morphology in frataxin-deficient glial cells and muscles. **(A)** Representative image of 35-day-old control GGCs (UAS-*ERGFP*/+; *Repo*-GAL4/+) stained with anti-Repo antibody to label cell nuclei. Perinuclear localization of ER cisternae is observed. **(B)** Representative image of 35-day-old frataxin-depleted GGCs (UAS-*ERGFP*/*fhRNAi-1*; *Repo*-GAL4/+) stained with anti-Repo antibody to label cell nuclei. No important alteration of the ER network was detected. **(C)** Representative image of 7-day-old control muscles (UAS-*ERGFP*/+; *Mef2*-GAL4/+) stained with phalloidin to label F-actin of muscle fibers. ER is organized as single signals in a ladder-like network along the muscle fibers with occasional aggregates of ERGFP signals. **(D)** Representative image of 7-day-old frataxin-depleted muscles (UAS-*ERGFP*/*fhRNAi-1*; *Mef2*-GAL4/+) stained with phalloidin to label F-actin of muscle fibers. The overall organization of ER cisternae is well conserved, although a strong increase of GFP positive clusters is present throughout the muscular system (arrows) disturbing the ER network. Scale bars represent 40 μm in **(A,B)** and 10 μm **(C,D)**.

FIGURE S6 *Mitofusin* silencing fails to improve frataxin deficiency in muscle cells. **(A)** *Marf* downregulation does not influence frataxin levels. Since targeted analysis of frataxin levels in glia are methodologically not possible (lack of appropriate antibodies to detect endogenous frataxin or the masking effects of neurons in a Real Time PCR approach), the experiments were performed in fly muscles. **(B)** Negative geotaxis performance of 5-day-old flies displaying muscle-specific frataxin silencing and coexpression of our transgene’s collection. Besides frataxin overexpression, none of the other potential interactors was able to significantly improve locomotion of FRDA flies. Remarkably, *Marf* silencing worsened the phenotype. Graphs represent means ± SEM. Data was analyzed by Unpaired *T* test in **(A)** and one-way ANOVA with *post hoc* Dunnett Multiple Comparison Test in **(B)**. **P* < 0.05; ****P* < 0.001.

FIGURE S7 Impact of hydrogen peroxide scavengers on FRDA phenotypes. **(A)** Locomotor performance of 10-day-old flies displaying glial-specific frataxin silencing and coexpression of antioxidant genes. **(B–D)** Representative ultrathin plastic epon section of a 35-day-old frataxin-deficient flies coexpressing a neutral transgene (**B**, *fhRNAi-1*/+; *Repo*-GAL4/UAS-*GFP*), mitochondrial catalase (**C**, *fhRNAi-1*/+; *Repo*-GAL4/*mitoCatalase*) or mitochondrial thioredoxreductase (**D**, *fhRNAi-1*/+; *Repo*-GAL4/UAS-*TrxmitoB*). No rescue of lipid accumulation (arrows) and brain vacuolization (asterisks) was detected in the two tested genotypes. In **(A)**, means ± SEM, and data was analyzed by one-way ANOVA with *post hoc* Dunnett Multiple Comparison Test. ***P* < 0.01; ****P* < 0.001. Scale bars represent 50 μm in all cases.

## Abbreviations

ATF6: activating transcription factor 6
DRG: dorsal root ganglia
DRP1: dynamin-related protein 1
eIF2α: α subunit of eukaryotic translation initiation factor 2
ER: endoplasmic reticulum
FLP/FRT: Flippase/Flippase Recognition Target
FRDA: Friedreich’s ataxia
GGCs: giant glial cells
IFM: Indirect Flight Muscles
IRE1: inositol-requiring enzyme 1
MAMs: mitochondria-associated membranes
mitoGFP: mitochondria-targeted GFP
OPA1: optic atrophy 1
PBA: 4-phenylbutyric acid
PERK: pancreatic ER kinase
TUDCA: tauroursodeoxycholic acid
TMRE: tetramethylrhodamine, ethyl ester
UPR: unfolded protein response.
