# Supplementary material for: Mitofusin-Dependent ER Stress Triggers Glial Dysfunction and Nervous System Degeneration in a Drosophila Model of Friedreich’s Ataxia
Source: Front Mol Neurosci. 2018 Mar 6;11:38. doi: 10.3389/fnmol.2018.00038 (PMC5845754; doi:10.3389/fnmol.2018.00038)
Supplement: Supplementary file 1 [file Data_Sheet_1.docx]

**Additional methods**

***Drosophila* Stocks**

**Table 1. List of stocks used in the manuscript**

| Name | Genotype | Kindly Provided |
| --- | --- | --- |
| *W1118* | w[1118] | Bloomington Stock 3605 |
| *Pink1B9* | w[*]Pink1[B9]/FM7i, P{w[+mC]=ActGFP}JMR3 | Prof. Alex Whitworth and Prof. Leo Pallanck |
| *actin*-GAL4 | y[1]w[*];P{w[+mC]=Act5C-GAL4}17bFO1/TM6B, Tb[1] | Bloomington Stock 3954 |
| *Repo*-GAL4 | w[1118]; P{w[+m*]=GAL4}repo/TM3, Sb[1] | Bloomington Stock 7415 |
| *Mef2*-GAL4 | y[1] w[*]; P{w[+mC]=GAL4-Mef2.R}3 | Bloomington Stock 27390 |
| *fhRNAi-1* | w[1118]; P{w[+mC]=UAS-fhRNAi-1} | Prof. John P. Phillips |
| *fhRNAi-2* | y[1] w[*]; P{w[+mC]= UAS-fhRNAi-2}2 | Prof. Maria D. Moltó |
| UAS-*nGFP* | P{w[+mC]=UAS-Stinger}2 | Bloomington Stock 64502 |
| UAS-*mitoGFP* | P{w[+mC]=UAS-mito-HA-GFP.AP}/CyO | Bloomington Stock 8442 |
| UAS-*GFP* | y[*] w[*]; P{w[+mC]=UAS-2xEGFP}AH3 | Bloomington Stock 6658 |
| UAS-*ERGFP* | w[*]; P{w[+mC]=UAS-GFP.KDEL}11.1 | Bloomington Stock 9898 |
| UAS-*Xbp1GFP* | w[*]; P{w[+mC]=UAS-Xbp1.EGFP.LG}2/CyO | Pedro Domingos |
| UAS-*LAMP1GFP* | w[1118]; UAS-LAMP1GFP/CyO; TM6/Sb | Fabio Demontis |
| UAS-*GFPATG8a* | w[1118]; P{w[+mC]=UAS- GFP.Atg8a}3 | Prof. Thomas P. Neufeld |
| UAS-*ATG8a* | w[1118]; UAS-ATG8a(13)/TM3 | Jose A. Botella |
| UAS-*ATG1* | [y[1] w[*]; P{w[+mC]=UAS-Atg1.S}6B](http://flybase.org/cgi-bin/fbidq.html?FBst0051655) | Bloomington Stock 51655 |
| UAS-*mcherryRNAi* | y[1] sc[*] v[1]; P{y[+t7.7] v[+t1.8]=UAS-mCherry.VALIUM10}attP2 | Bloomington Stock 35787 |
| UAS-*fh* | y[1]w[*];P{w[+mC]=UAS- fh}2 | Prof. Maria D. Moltó |
| UAS-*FXN* | yw; UAS-FXN /TM3 | Prof. Maria D. Moltó |
| UAS-*MarfRNAi* | UAS-*MarfRNAi* /TM2 | Prof. Ming Guo |
| UAS-*MarfRNAi* (TRiP) | y[1] sc[*] v[1]; P{y[+t7.7] v[+t1.8]=TRiP.HMC03883}attP40 | Bloomington Stock 55189  Valium 20 |
| UAS-*Marf* | w[*]; UAS-Marf (3M) /TM3 | Prof. Alex Whitworth and Prof. Leo Pallanck |
| UAS-*Opa1RNAi* | UAS-*Opa1RNAi* /TM2 | Porf. Ming Guo |
| UAS-*Opa1* | w[*]; UAS-Opa1 (1) /CyO | Prof. Alex Whitworth and Prof. Leo Pallanck |
| UAS-*drp1RNAi* | y[1]v[1];P{y[+t7.7] v[+t1.8]=TRiP.HMC03230} attP40 | Bloomington Stock 51483  Valium 20 |
| UAS-*drp1* | UAS-*drp1*/TM6C | Prof. Ming Guo |
| UAS-*parkinRNAi* | y[1] sc[*] v[1]; P{y[+t7.7] v[+t1.8]=TRiP.HMS01800}attP2/TM3, Sb[1] | Bloomington Stock 38333  Valium 20 |
| UAS-*parkin* | w[*]; UAS-park (C2) (II) | Prof. Alex Whitworth and Prof. Leo Pallanck |
| UAS-*pinkRNAi* | y[1] sc[*] v[1]; P{y[+t7.7] v[+t1.8]=TRiP.HMC04160}attP2 | Bloomington Stock 55886  Valium 20 |
| UAS-*Pink1* | w[*]; UAS-HA-Pink1 / CyO | Prof. Alex Whitworth and Prof. Leo Pallanck |
| UAS-*SpargelRNAi* | y[1] sc[*] v[1]; P{y[+t7.7] v[+t1.8]=TRiP.HMS00857}attP2 | Bloomington Stock 33914  Valium 20 |
| UAS-*Spargel* | Yw hs flp; UAS-Spargel / CyO | Prof. Christian Frei |
| UAS-*delg* | yw; UAS-delg-HA / CyO | Prof. Hugo Stocker |
| UAS-*MiroRNAi* | y[1] v[1]; P{y[+t7.7] v[+t1.8]=TRiP.JF02775}attP2/TM3, Sb[1] | Bloomington Stock 27695  Valium 10 |
| UAS-*Miro* | w[*]; P{w[+mC]=UAS-Miro.G}2 | Prof. Konrad Zinsmaier |
| UAS-*MiltonRNAi* | y[1]v[1];P{y[+t7.7] v[+t1.8]=TRiP.HMC02365 }attP2 | Bloomington Stock 44477  Valium 20 |
| UAS-*KhcRNAi* | y[1] sc[*] v[1]; P{y[+t7.7] v[+t1.8]=TRiP.HMS01519}attP2 | Bloomington Stock 35770  Valium 20 |
| *mitoCatalase* | P{OAT-Cat}2d5a | Prof. William C. Orr and Robin J. Mockett |
| *C7* | P{ }C7. Transgenic Control insertion for *mitoCatalase* | Prof. William C. Orr and Robin J. Mockett |
| UAS-*Sod2* | w[1]; P{w[+mC]=UAS-Sod2.M}UM83 | Fanis Missirlis |
| UAS-*TrxRmitoB* | w[1118]; P{w[+mC]=UAS-TrxR-1^mito^} | Fanis Missirlis |
| Integration cassette ΦX-86Fb | yw; M{eGFP.vas-int.Dm}ZH-2A; +; M{RFP.attP}ZH-86Fb; + | Prof. Michael Krahn |
| control | [y[1] w[1118] P{ry[+t7.2]=neoFRT}19A](http://flybase.org/cgi-bin/fbidq.html?FBst0001744) | Bloomington Stock 1744 |
| *fh^1^* | y[1] w[*] fh[1] P{ry[+t7.2]=neoFRT}19A/FM7c, P{w[+mC]=GAL4-Kr.C}DC1, P{w[+mC]=UAS-GFP.S65T}DC5, sn[+] | Bloomington Stock 67161 |
| *ey-GAL4 UAS-FLP* | P{w[+mC]=GMR-hid}SS1, y[1] w[*] P{ry[+t7.2]=neoFRT}19A, l(1)CL[1]/FM7a; P{w[+m*]=GAL4-ey.H}SS5, P{w[+mC]=UAS-FLP.D}JD2 | Bloomington Stock 5249 |

**Real-Time PCR**

**Table 2. List of primers used in the Real Time PCR experiments.**

| *Gene* | Primer sequence |
| --- | --- |
| *BiP*  (CG4147) | Fw: 5’-TGTCACCGATCTGGTTCTTCAGGC-3’  Rv: 5’-GTCCCATGACCAAGGACAACCATC-3’ |
| *Drp1*  (CG3210) | Fw: 5’-GGACAAGAATCTGGATGAGGTC-3’  Rv: 5’-CGCTATGACCTCCAGTTGC-3’ |
| *FH*  (CG8971) | Fw: 5’-GTCACAGTCCGTGGACTTCC-3’  Rv: 5’-CAAAATCGAACGTTTCAACCG-3’ |
| *Mfn/Marf*  (CG3869) | Fw: 5’-ACCTCACCTCGGCCAACT-3’  Rv: 5’- GTGGTGGCGGTATCAACC-3’ |
| *Opa1*  (CG8479) | Fw: 5’-TGCACAGTCAGGTTCTCAAAA-3’  Rv: 5’-TATGAATTCCTGCTGCAACG-3’ |
| *Parkin*  (CG10523) | Fw: 5’-GCCTGCACGGATGTGAGT-3’  Rv: 5’- AAACGGGAACGGCAATAAT-3’ |
| *Pink1*  (CG4523) | Fw: 5’-TCGACGATTTCGCCTTGTA-3’  Rv: 5’-GTGGTGGTTGTGGTGCAG-3’ |
| *Ref(2)P/p62*  (CG10360) | Fw: 5’-CGTAAGGACCTTCTGGATCG-3’  Rv: 5’-GTGCATATTGCTCTCGCACT-3’ |
| *RP49*  (CG7939) | Fw: 5’-CCAAGCACTTCATCCGCCACC-3’  Rv: 5’-GCGGGTGCGCTTGTTCGATCC-3’ |
| *Spargel*/*Srl*  (CG9809) | Fw: 5’-TCTGCCCTATGGAAGCATTAAAC -3’  Rv: 5’-CATACATGCTTATTTGCGAGTCTCT -3’ |
| *Tfam*  (CG4217) | Fw: 5’-CACCTCGACGGTGGTAATCT-3’  Rv: 5’-AAGACCCTGGAGGAGCAGTT-3’ |

**Confocal microscope settings and Image handling**

Scan speed, amplifier gain and pinhole size were kept constant across all images. When LSM 510 was used, samples were excited with an argon laser at 488 nm (average tube current: 6.2 A and 25% trans-mission) and a helium-neon laser at 543 nm (100% transmission). Emitted signals were detected with a Plan-Neofluar 20x/0.5 and Plan-Apochromat 63x/1.4 objectives at 505-530nm (GFP) and >560nm (Cy3) at a resolution of 1024 x 1024 pixels. In case of TSC SP8, samples were excited with a UV diode at 405nm, an argon laser at 488 nm (10% tube current), a DPSS laser at 561 nm and a helium-neon laser at 594 nm. Emitted signals were detected with a HC PL APO 40x/1.30 Oil CS2 objective (brains) or HC PL APO 63x/1.30 Glyc CORR CS2 objective (muscles) at 410-470nm (DAPI), 490-540nm (GFP), >565nm (AF555), >640nm (AF633). Images were generated at a resolution of 1024 x 1024 pixels. Brains and muscles were scanned in z-stacks (1 µm and 0.33 µm, respectively) with 30-35 images per brain/muscle.

For the quantification of number and size of p62-positive vesicles in Additional file 4 and colocalization analysis in Figure 4, five thoraxes per genotype and condition were analyzed. For each thorax, five independent areas of 1600 µm^2^ were selected and a z-projection of 30 single images was generated (diameter: 10 µm). In each random square of 1600 µm^2^, the threshold was set using the default method in ImageJ. The particles were then analyzed with the following options: Size 30-Infinity, Circularity 0.40-1.00, Include holes. The following parameters were measured: Count; Average Size. The resulting values were averaged.
