# Supplementary material for: Mitofusin-Dependent ER Stress Triggers Glial Dysfunction and Nervous System Degeneration in a Drosophila Model of Friedreich’s Ataxia
Source: Front Mol Neurosci. 2018 Mar 6;11:38. doi: 10.3389/fnmol.2018.00038 (PMC5845754; doi:10.3389/fnmol.2018.00038)
Supplement: Supplementary file 2 [file Data_Sheet_2.pdf]

Figure 2G

Blot used to quantify p62 levels in 35-day-old brains. The broken line denotes the areas shown in Figure 2G

The information about sizes from New England Biolabs (product P7709) has been incorporated to facilitate size comparison.

Fly p62 runs about 100KDa and the loading control tubulin around 52-55KDa

Repo-GAL4>*UAS-mcherryRNAi:* Samples 2-4-6

Repo-GAL4>*fhRNAi-1:* Samples 1-3-5

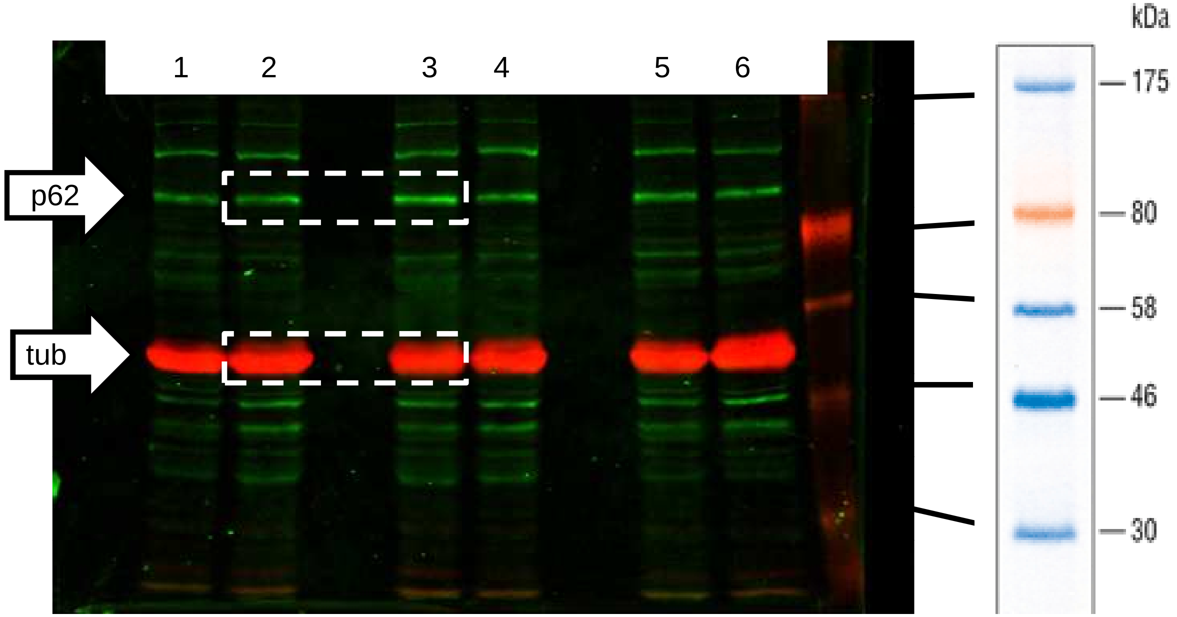

Figure 3E

Representative blot used to quantify p62 levels in 5-day-old thoraces.
The signals corresponding to p62 in frataxin-deficient flies were so strong that the intensity of both channels was reduced for proper quantification and under these settings the molecular weight marker was not longer visible.
The broken lines denote the areas plotted in Figure 3E.

The panel down shows the same western blot picture but with enhanced brightness and contrast to allow partial visualization of the molecular weight marker. The information about sizes from New England Biolabs (product P7709) has been incorporated to facilitate size comparison.

Fly p62 runs about 100KDa and the loading control tubulin around 52-55KDa

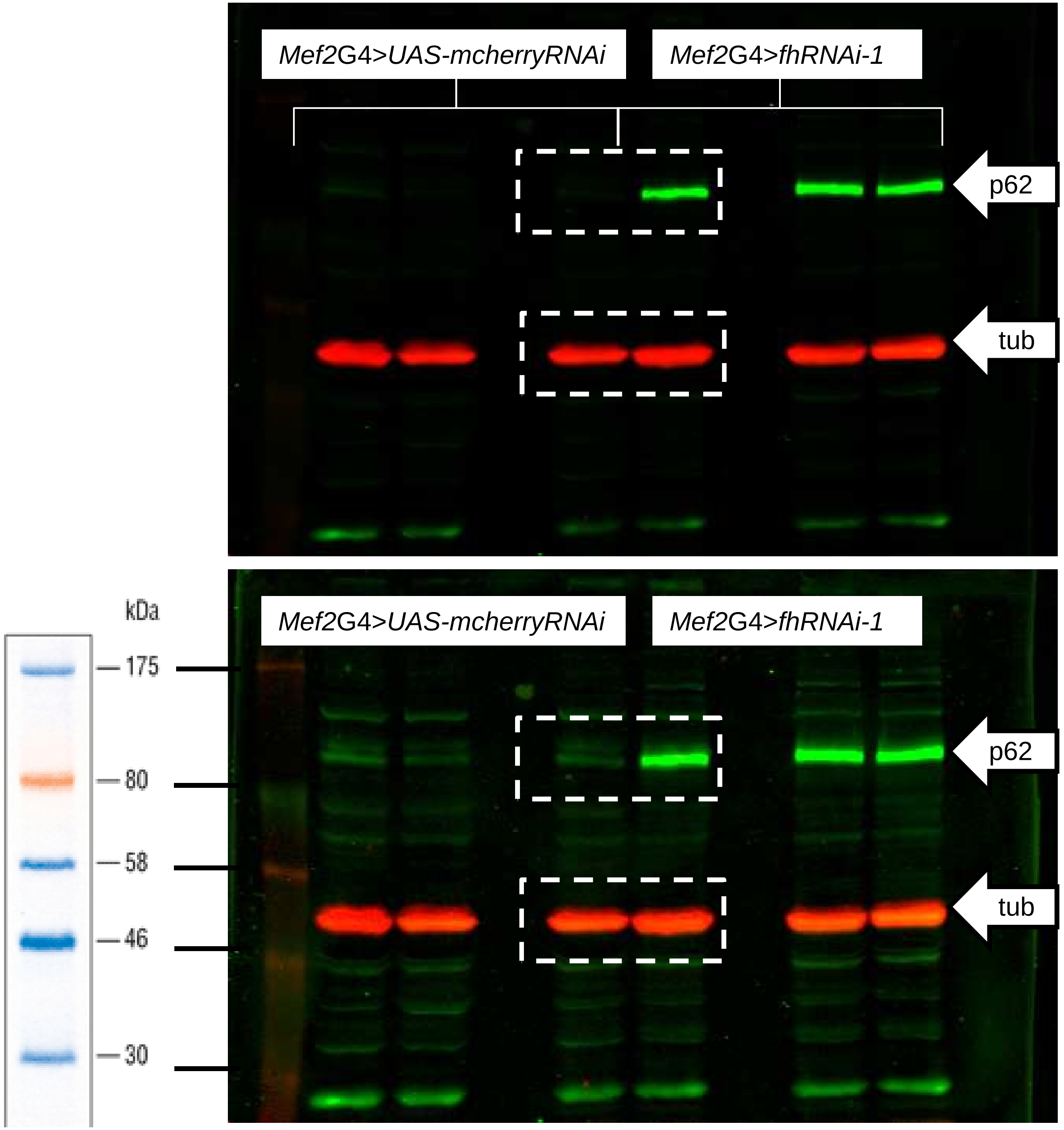

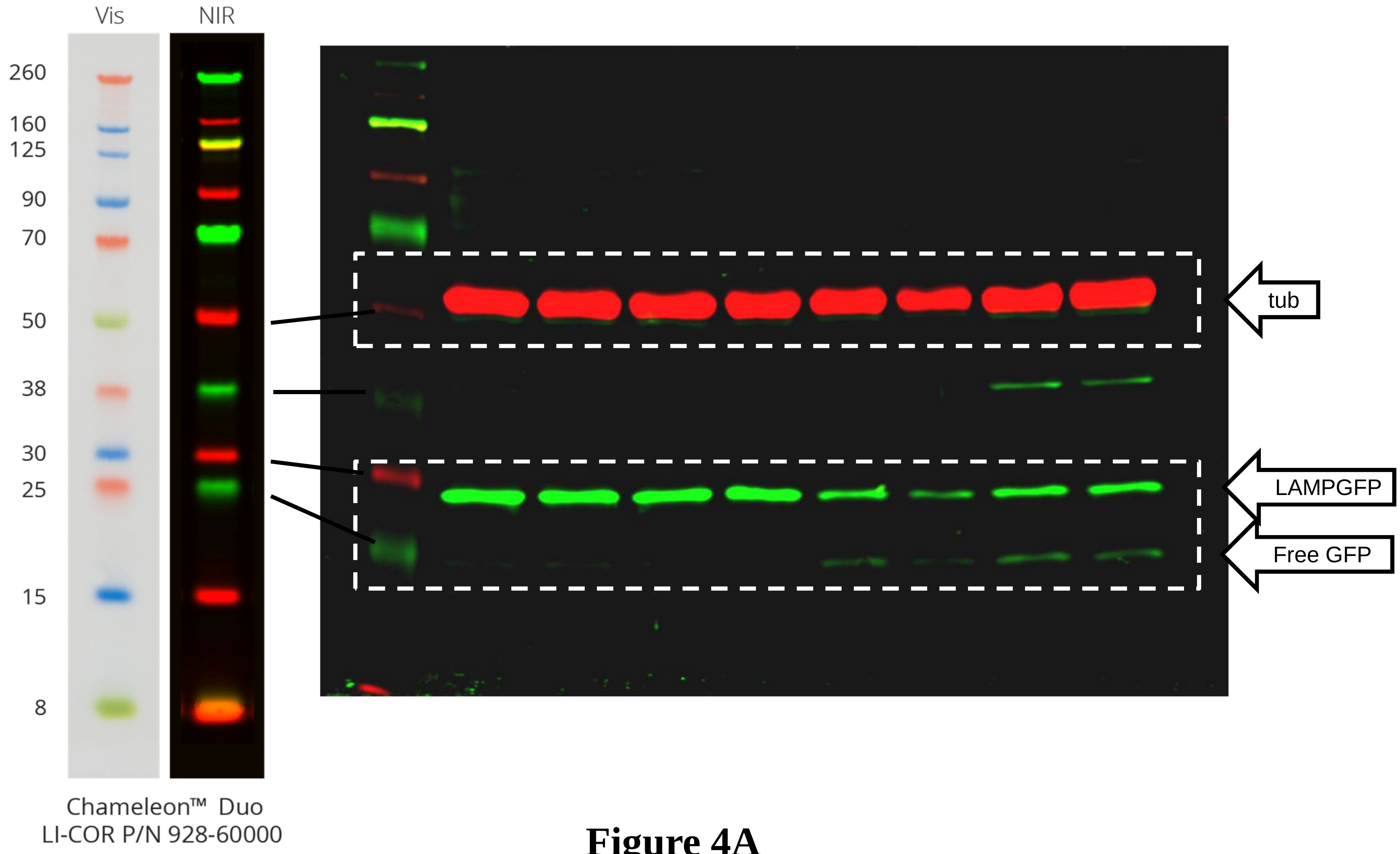

Figure 4A

Blot used to quantify LAMP1GFP and Free GFP amounts in controls and frataxin deficient glia shown in Fig 4B-C. The broken line denotes the areas shown in Figure 4A. The information about sizes from the manufacturer Li-Cor has been also added to facilitate comparison. Detection of LAMP1GFP (Green band of approximately 30KDa) and Free GFP (Green band of approximately 20KDa) levels in controls and FRDA brain samples using an anti-GFP antibody. Tubulin (red band of 55KDa) was used as loading control.

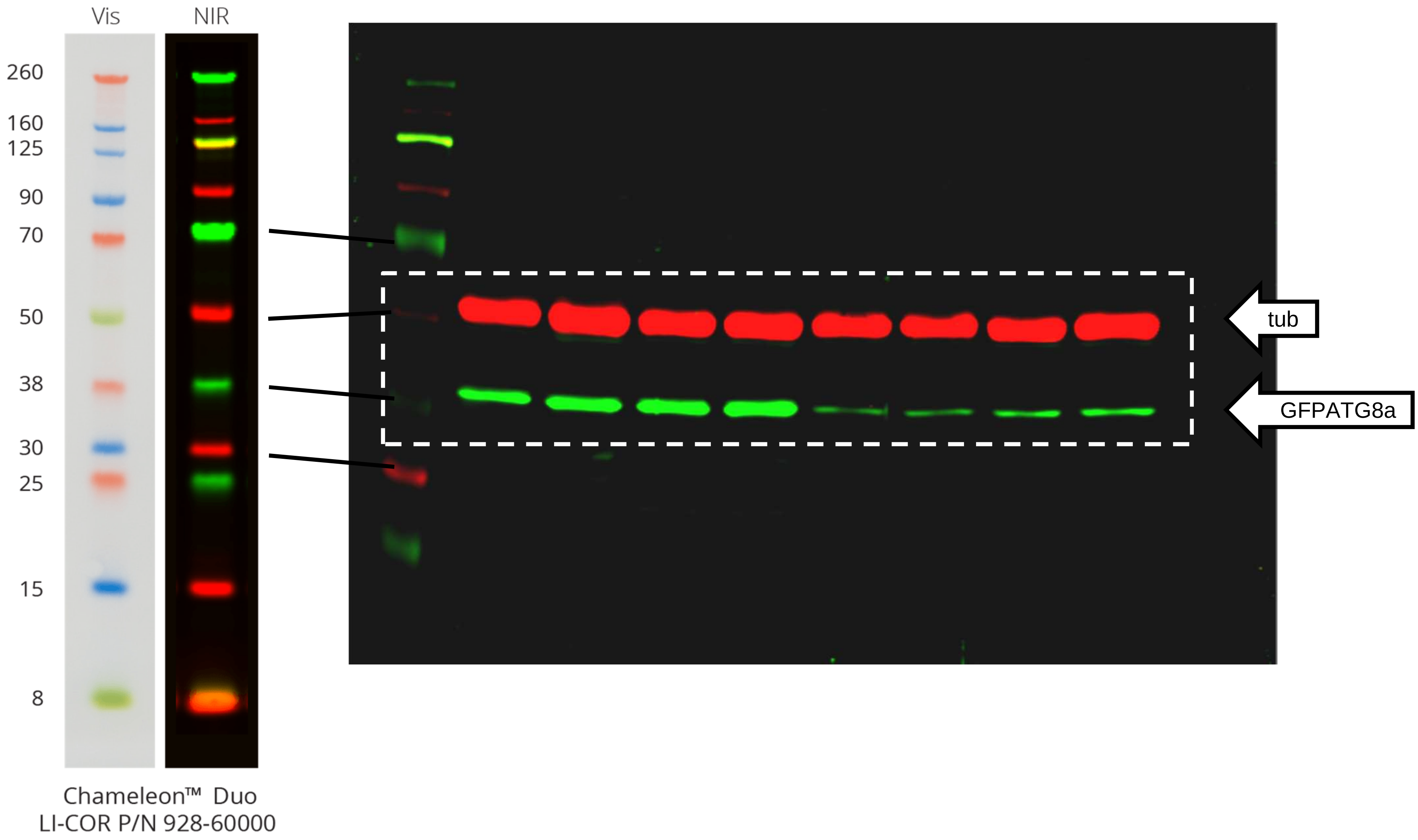

Figure 4A

Blot used to quantify GFPATG8a amounts in controls and frataxin deficient brains in Figure 4B. The broken line denotes the areas shown in Fig 4A. The information about sizes from the manufacturer Li-Cor has been also added to facilitate comparison. Detection of GFPATG8a (Green band of approximately 45KDa). Tubulin (red band of 55KDa) was used as loading control.

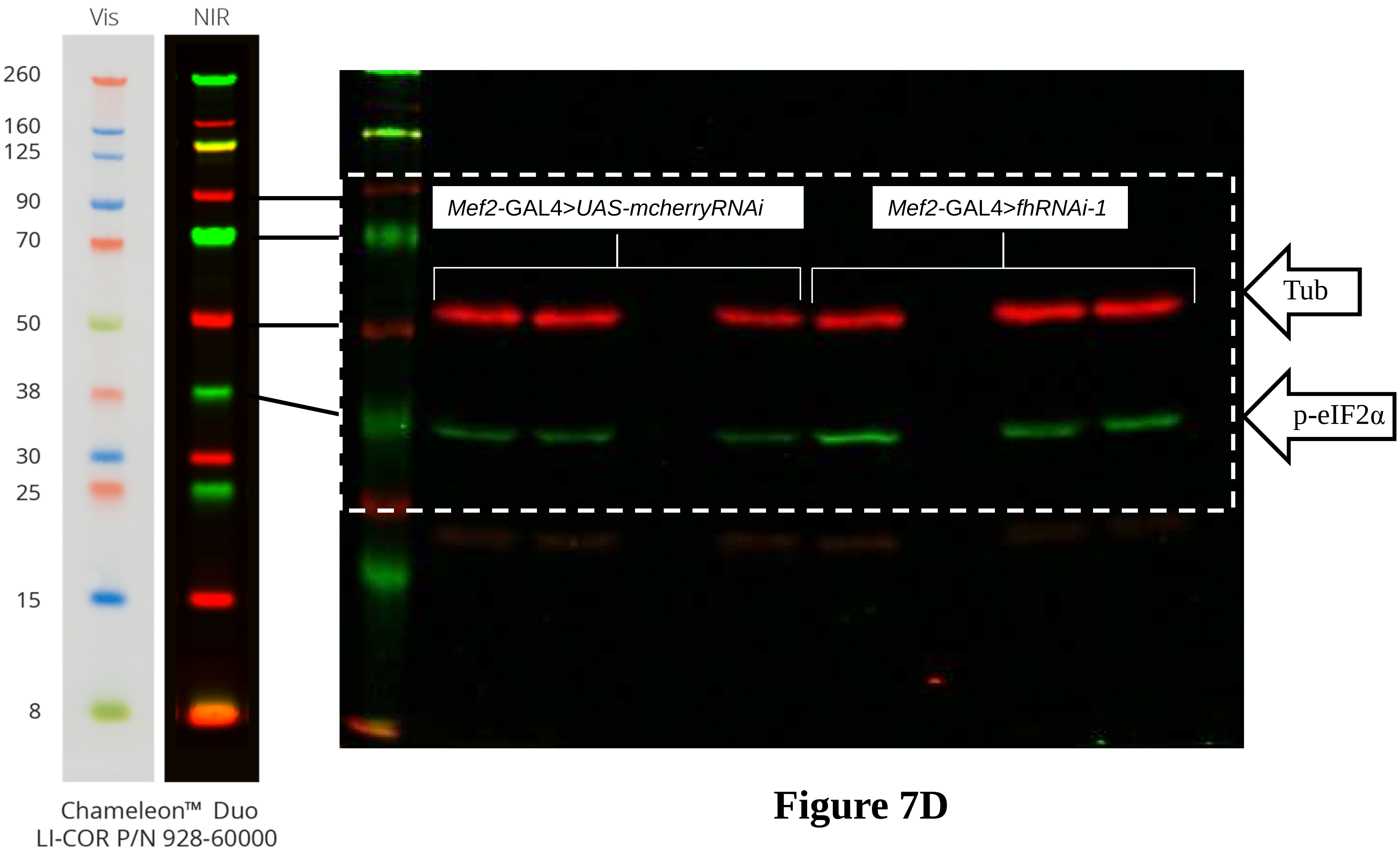

Figure 7D

Blot used to quantify peIF2α levels in controls and frataxin deficient muscles shown in Figure 7D. The broken line denotes the area shown in Figure 7D. The information about sizes from the manufacturer Li-Cor has been also added to facilitate comparison. Fly peIF2α runs about 38KDa (green band) and the loading control tubulin around 52-55KDa (red band).

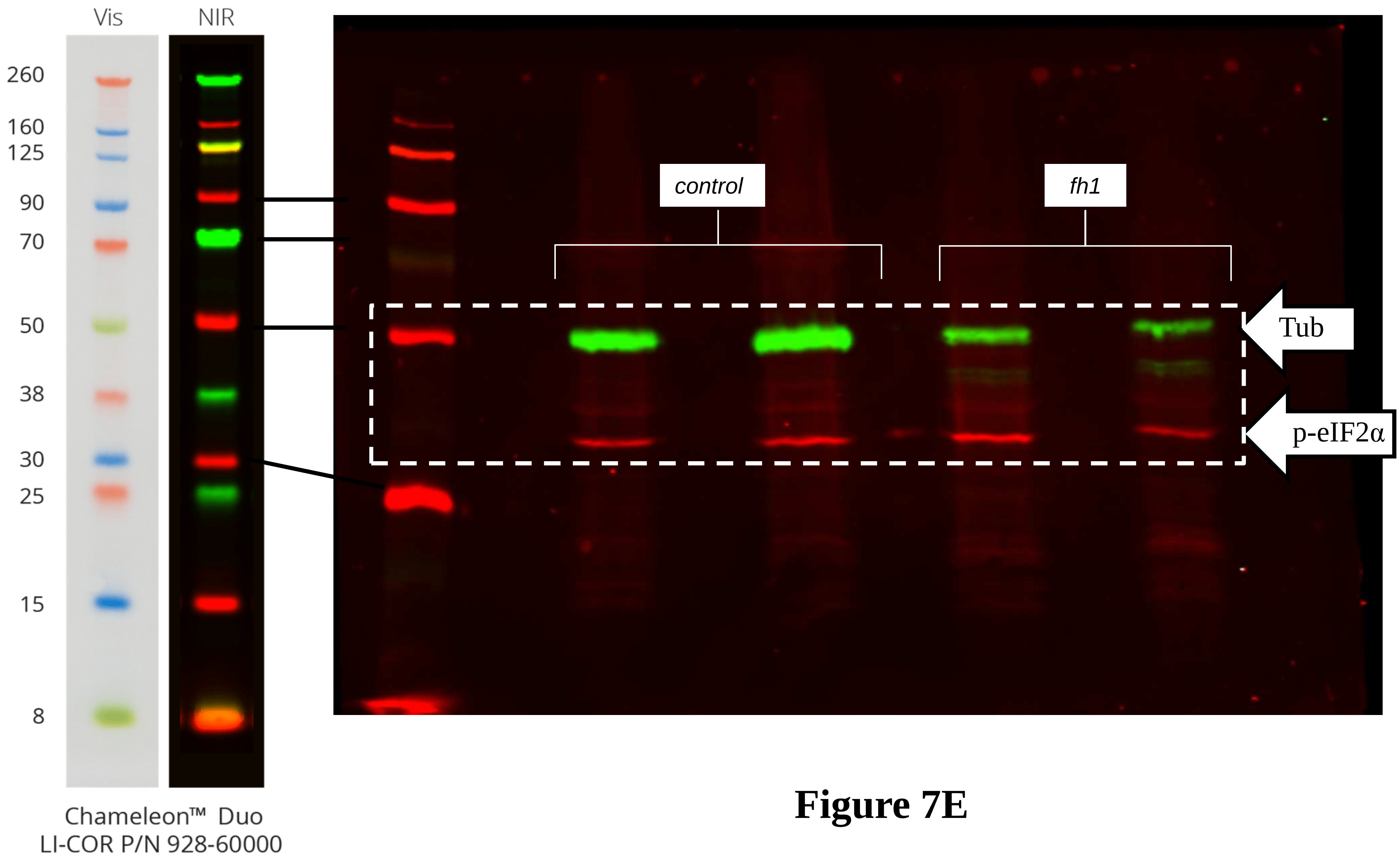

Figure 7E

One Representative Blot (from 2) used to quantify p-eIF2α levels in controls and frataxin mutant (fh1) larvae shown in Figure 7E. The broken line denotes the area shown in Figure 7E. The information about sizes from the manufacturer Li-Cor has been also added to facilitate comparison. Fly peIF2α runs about 38KDa (red band) and the loading control tubulin around 52-55KDa (green band).

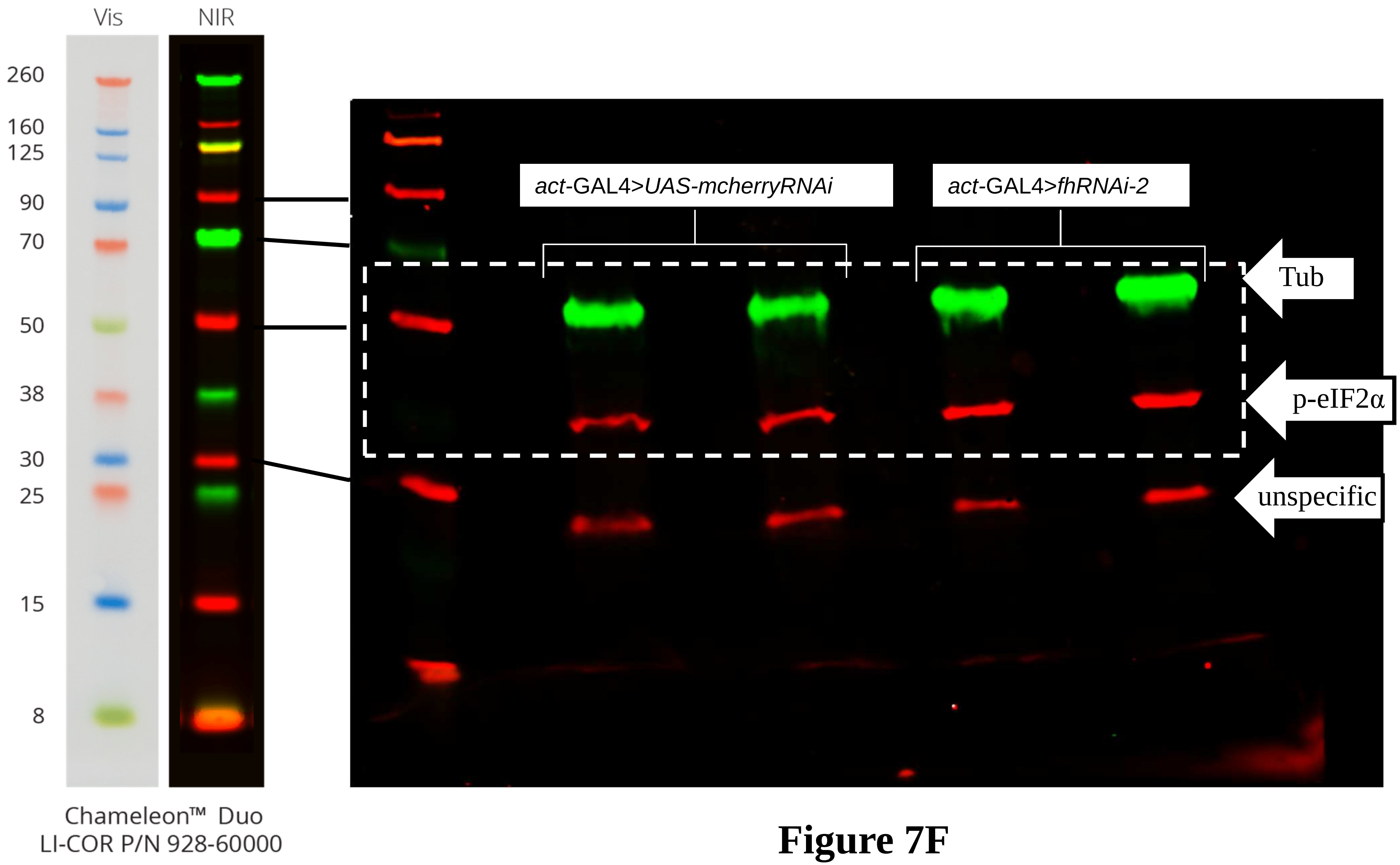

Figure 7F

One Representative Blot (from 3) used to quantify peIF2α levels in controls and frataxin deficient flies shown in Figure 7F. The information about sizes from the manufacturer Li-Cor has been also added to facilitate comparison. Fly peIF2α runs about 38KDa (red band) and the loading control tubulin around 52-55KDa (green band).

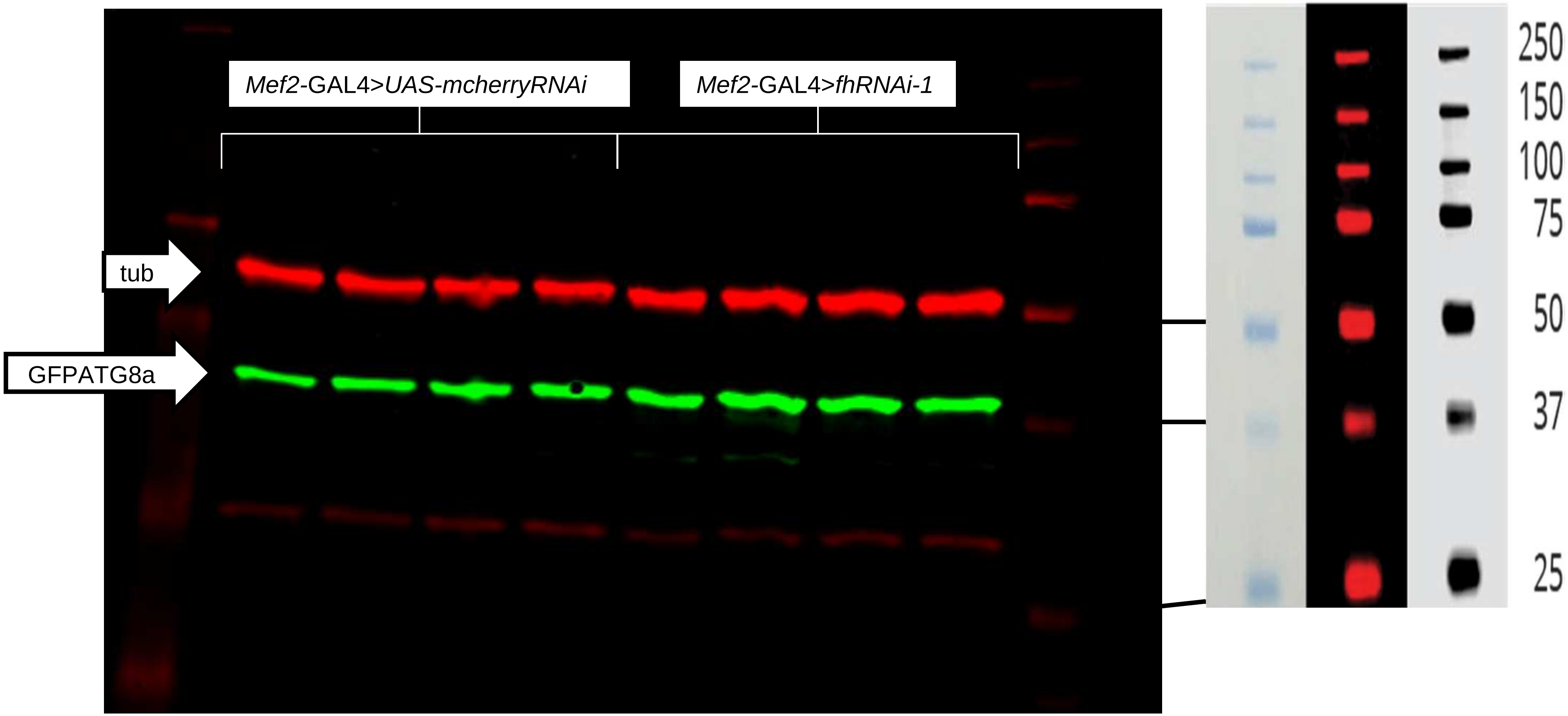

Supplementary Figure S3E

Blot used to quantify GFPATG8a amounts in controls and frataxin deficient muscles in Supplementary Figure S3E. The information about sizes from the manufacturer Li-Cor has been also added to facilitate comparison. Detection of GFPATG8a (Green band of approximately 45KDa). Tubulin (red band of 55KDa) was used as loading control.

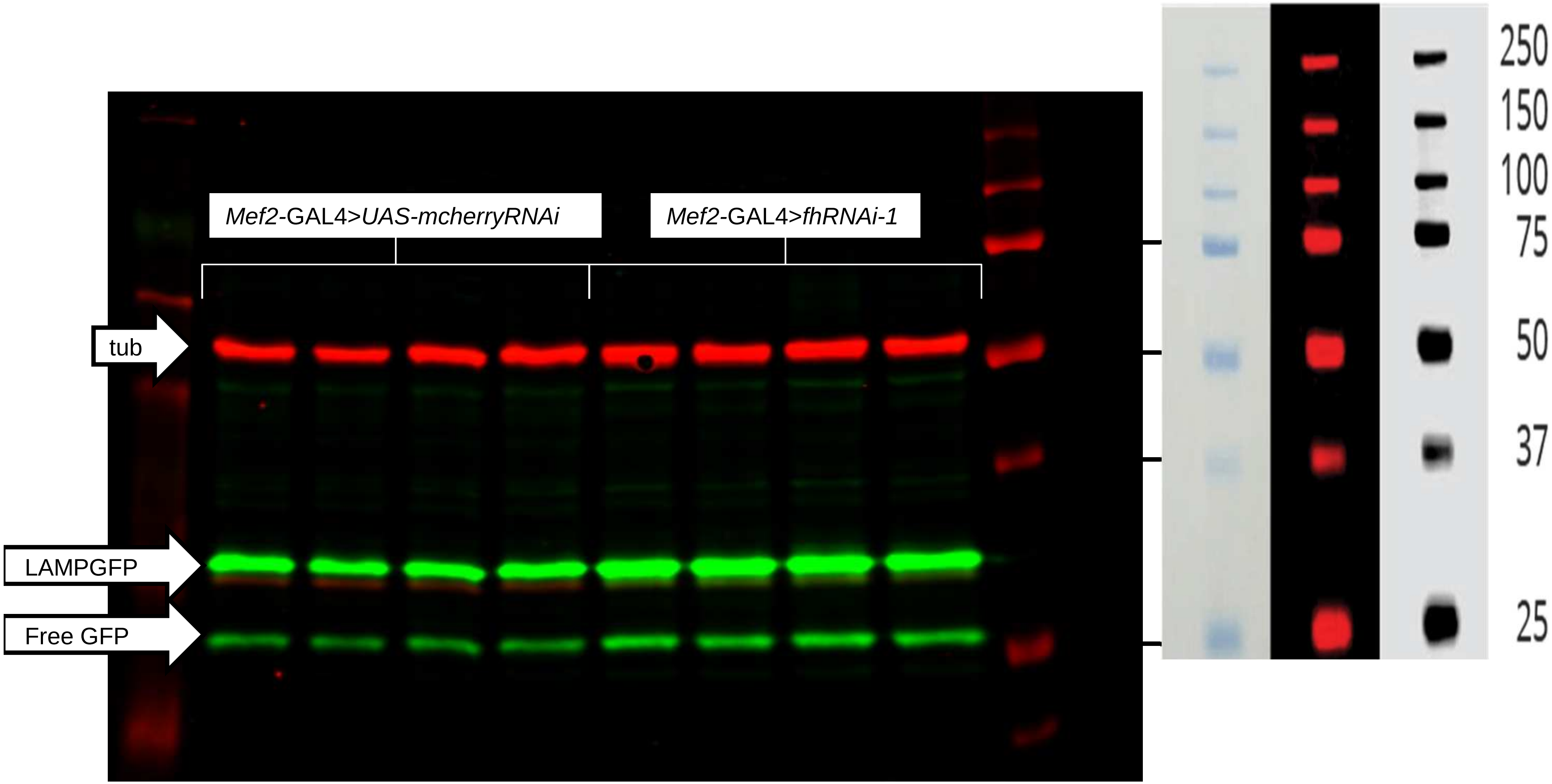

Supplementary figure S3E

Blot used to quantify LAMP1GFP and Free GFP amounts in controls and frataxin deficient muscle shown in Supplementary Figure S3E. The information about sizes from the manufacturer Li-Cor has been also added to facilitate comparison. Detection of LAMP1GFP (Green band of approximately 30KDa) and Free GFP (Green band of approximately 25KDa) levels in controls and FRDA brain samples using an anti-GFP antibody. Tubulin (red band of 55KDa) was used as loading control.

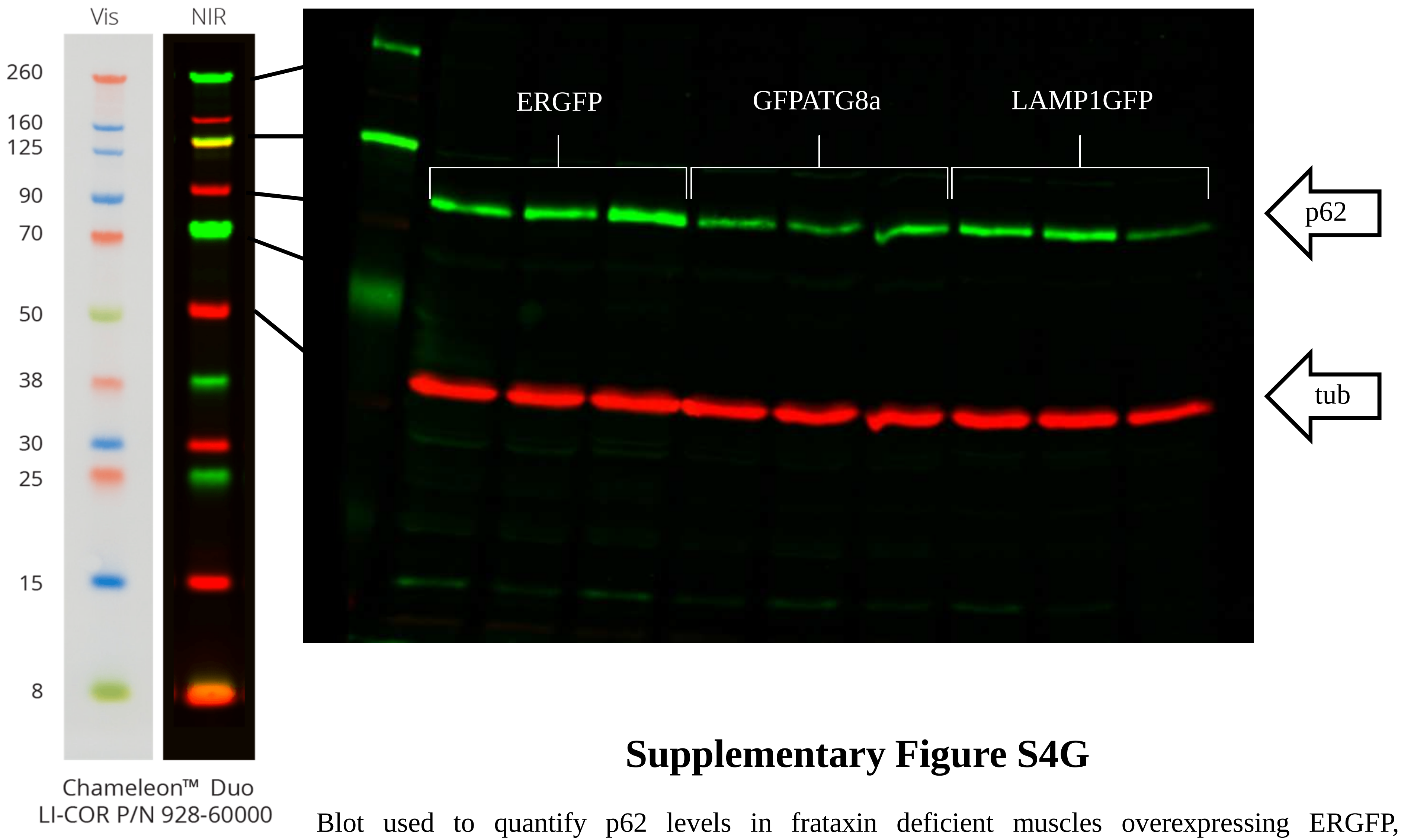

Supplementary Figure S4G

Blot used to quantify p62 levels in frataxin deficient muscles overexpressing ERGFP, GFPATG8a and LAMP1GFP in Supplementary Figure S4G. The information about sizes from the manufacturer Li-Cor has been also added to facilitate comparison. Fly p62 runs about 100KDa (green band) and the loading control tubulin around 52-55KDa (red band).
