# Supplementary figures and images for: Mitofusin-Dependent ER Stress Triggers Glial Dysfunction and Nervous System Degeneration in a Drosophila Model of Friedreich’s Ataxia
Source: Front Mol Neurosci. 2018 Mar 6;11:38. doi: 10.3389/fnmol.2018.00038 (PMC5845754; doi:10.3389/fnmol.2018.00038)

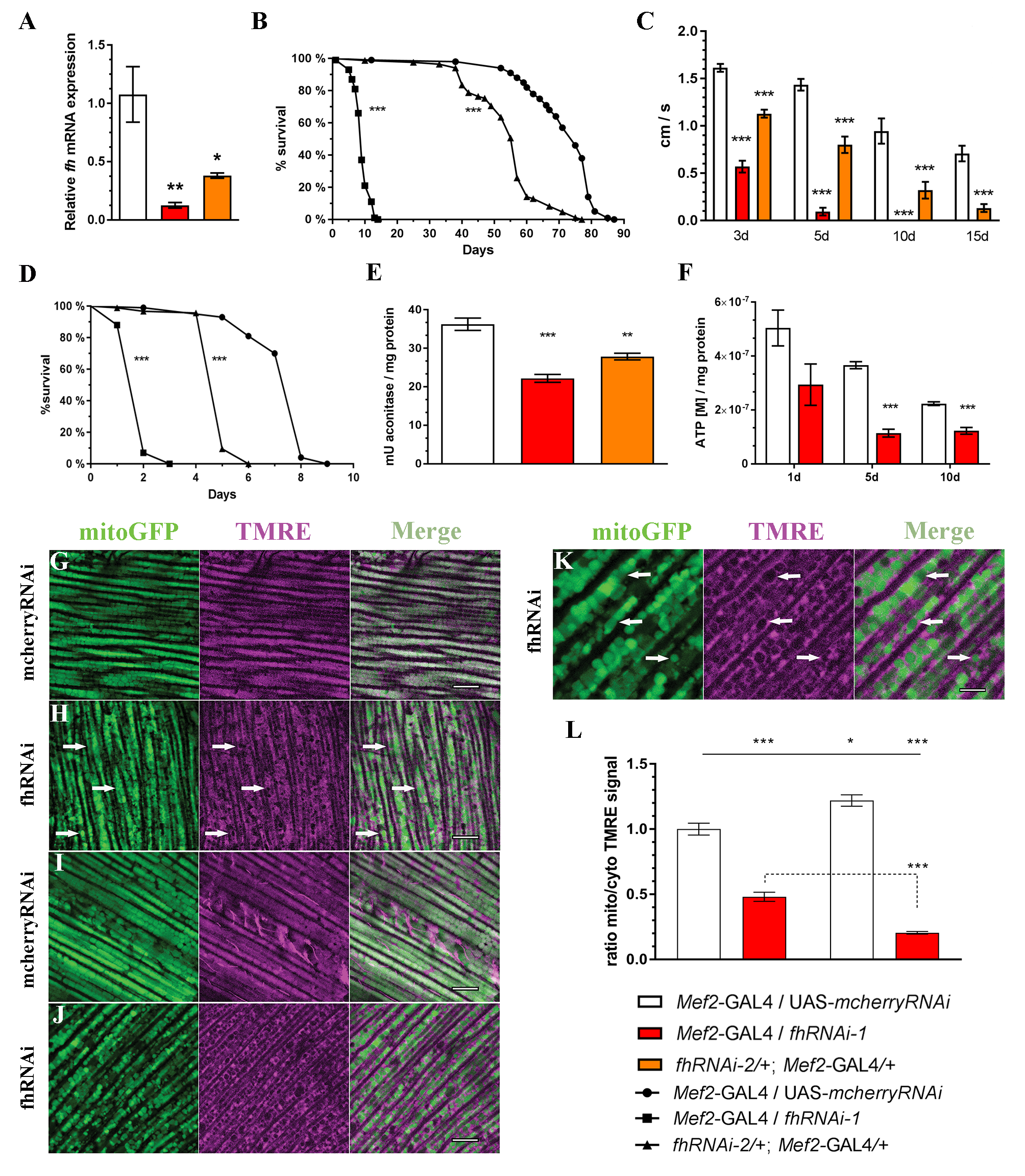

Supplement: FIGURE S1 — Behavioral and mitochondrial defects triggered by frataxin downregulation in fly muscles. (A) Two independent RNAi lines induce a frataxin downregulation in thoracic muscles using Mef2-GAL4 from 60% (fhRNAi-2) to 90% (fhRNAi-1). (B) Targeted frataxin knockdown in muscles was sufficient to shorten mean and maximum life span. (C) Frataxin silencing in muscles impairs negative geotaxis already at 3 days after eclosion and completely abolished locomotion after 5 days upon severe downregulation and after 15 days upon moderate silencing. (D) Frataxin depletion in the fly musculature induces hypersensitivity towards oxidative insult. (E) Aconitase activity is clearly diminished in frataxin-deficient muscles 5 days post eclosion. (F) ATP production is severely affected in frataxin-deficient flies (50%) and the age-dependent decay of ATP is enhanced upon frataxin-silencing. (G–J) In order to analyze individual differences between organelles, we stained the mitochondria with the permeable dye tetramethylrhodamine, ethyl ester (TMRE), which only accumulates inside of mitochondria, which are active and which have a normal membrane potential. (G) TMRE staining of 1-day-old control muscles. Perfect colocalization between mitoGFP and TMRE signals indicating functional mitochondria. (H) TMRE staining of 1-day-old Friedreich’s ataxia (FRDA) muscles. Arrows indicate lack of colocalization between mitoGFP and TMRE signals in some organelles suggesting impaired mitochondrial membrane potential already in young flies. (I) TMRE staining of 7-day-old control muscles. Perfect colocalization between mitoGFP and TMRE signals. (J) TMRE staining of 7-day-old FRDA muscles. No colocalization between mitoGFP and TMRE signals in most mitochondria indicating that membrane potential is abolished. (K) Magnification from (J). TMRE staining of 7-day-old FRDA muscles. Arrows denote representative mitochondria in which TMRE is not incorporated into the organelle due to a deficient membrane potential indi [file Image_1.TIF]

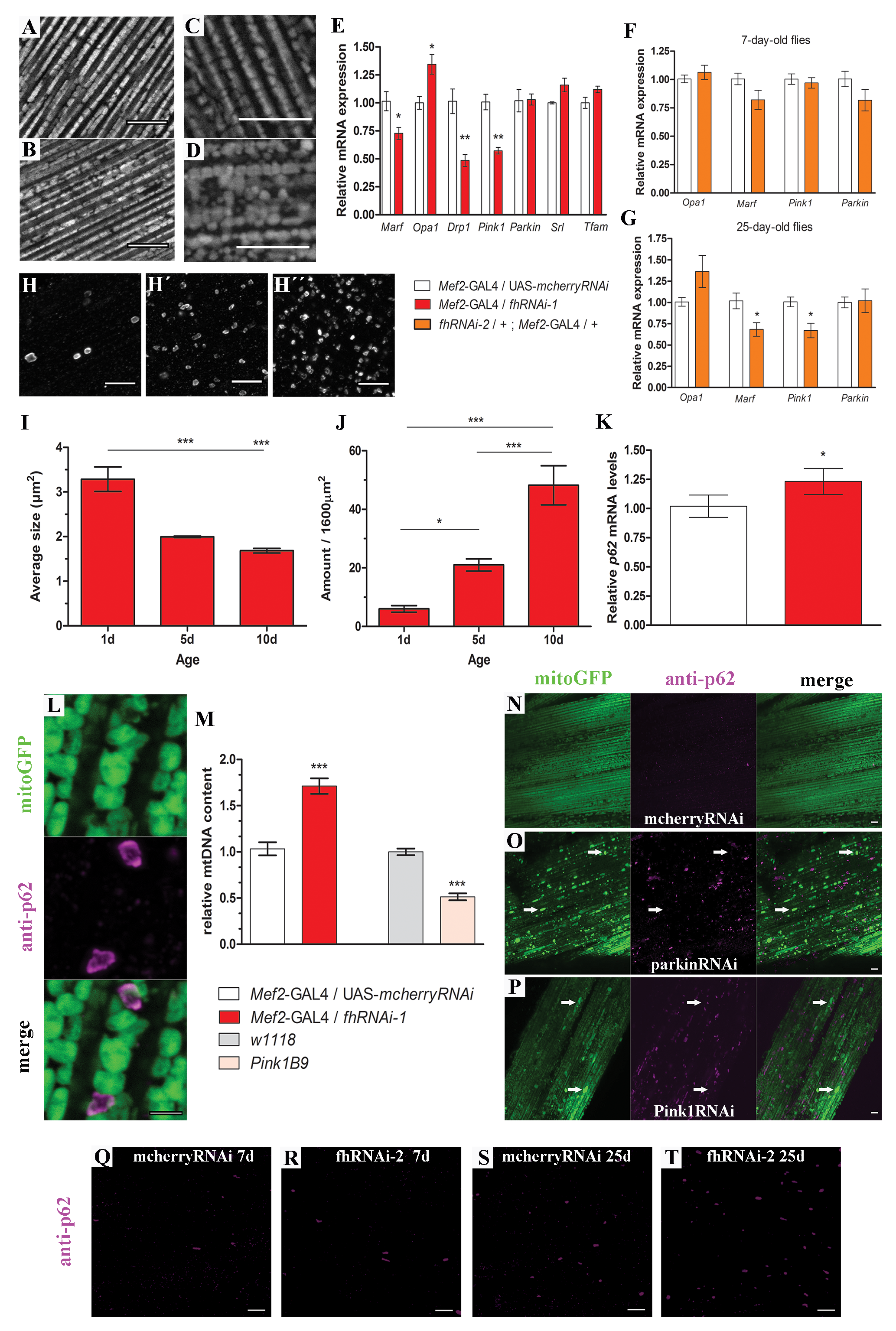

Supplement: FIGURE S2 — Analysis of age-dependent accumulation of p62-positive vesicles upon frataxin silencing in fly muscles. (A) MitoGFP labeled mitochondria from 1-day-old controls (UAS-mitoGFP/+; Mef2-GAL4/UAS-mcherryRNAi). (B) MitoGFP labeled mitochondria from 1-day-old frataxin-deficient thoraces (UAS-mitoGFP/+; Mef2-GAL4/fhRNAi-1). (C) MitoGFP labeled mitochondria from 25-day-old controls (UAS-mitoGFP/+; Mef2-GAL4/UAS-mcherryRNAi). (D) MitoGFP labeled mitochondria from 25-day-old frataxin-deficient thoraces (UAS-mitoGFP/fhRNAi-2; Mef2-GAL4/+) A second RNAi also induces some mitochondrial fragmentation but only in old flies. (E–G) Relative expression levels of genes involved in mitochondrial quality control and dynamics. Our results showed that Opa1 (involved in fusion) was upregulated and dynamin-related protein 1 (Drp1) (involved in fission) was clearly downregulated. Surprisingly, and in contrast to Opa1, Drosophila Mitofusin (Marf), a second gene involved in mitochondrial fusion, was slightly downregulated. In muscles, Marf also seems to behave differently to its counterparts in fusion and fission of mitochondria. Pink1 expression was reduced around 50% whereas the expression of parkin, Pink1’s partner in the activation of mitophagy, was not affected. We also found that the transcriptional coactivator spargel (Srl) and the transcription factor tfam, both involved in the biosynthesis of mitochondria, were not altered in the FRDA muscle cells. Similar results were obtained in old fhRNAi-2 flies. (H–H″) Representative pictures of 1600 μm2 from 1 (H), 5 (H′) and 10 (H″) day-old frataxin-deficient muscles stained with anti-P62 and used to calculate the results represented in (I,J). (I) Analysis of size of p62 vesicles in frataxin-deficient flies at three different time points (1, 5 and 10-day-old flies). We observed a reduction around 30% after 5 days and 50% after 10 days. This result is consistent with the increased mitochondrial fragmentation reported in Figure 3B. (J) Quantificat [file Image_2.TIF]

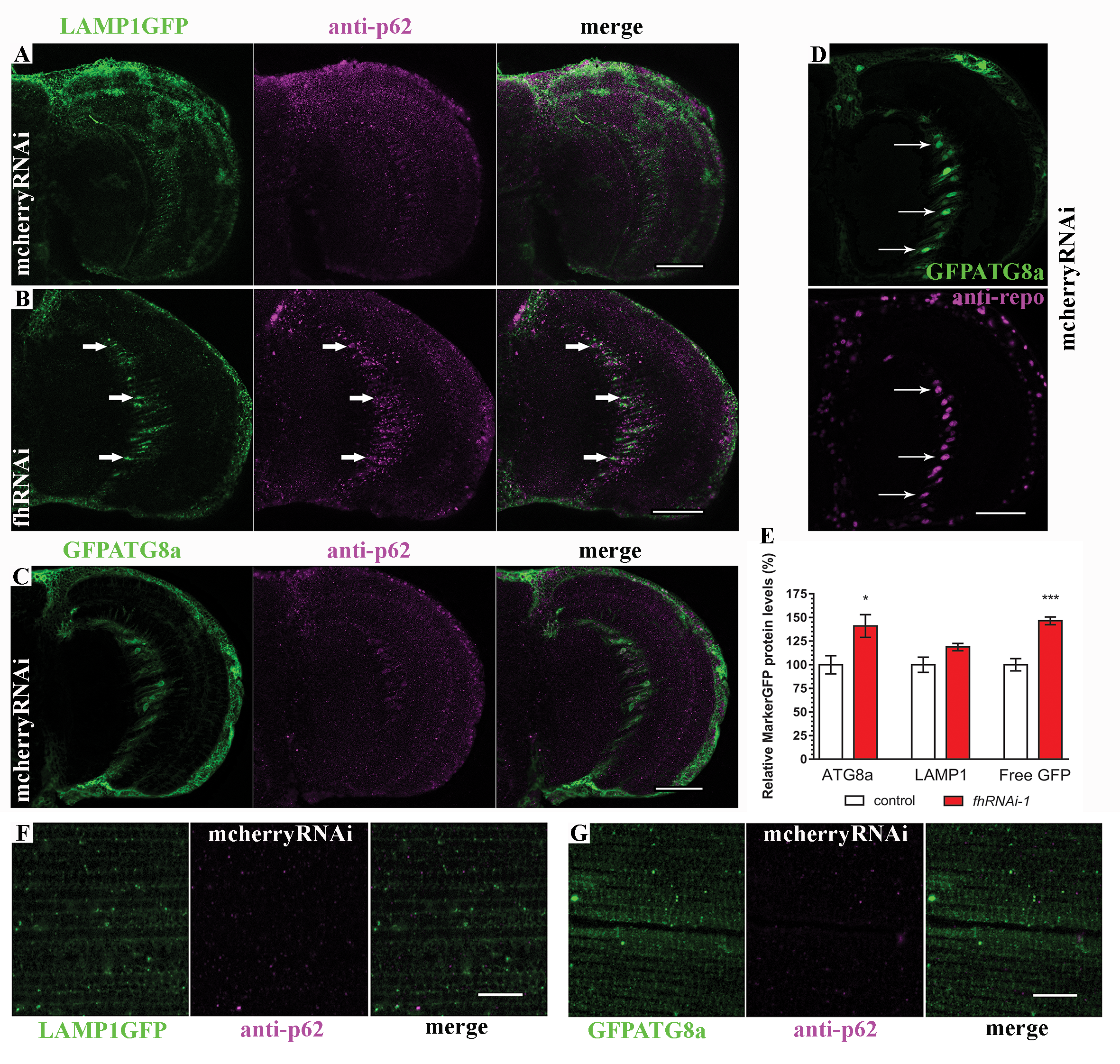

Supplement: FIGURE S3 — Histological analysis of autophagy markers in control flies. (A) Representative LAMP1GFP and p62 patterns in the GGCs of a 35-day-old control fly (UAS-LAMP1GFP/+; Repo-GAL4/UAS-mcherryRNAi). Controls presented little amounts of LAMP1 and p62 structures, indicative of basal autophagic levels. (B) Representative LAMP1GFP and p62 patterns in the GGCs of a 35-day-old FRDA fly (UAS-LAMP1GFP/+; Repo-GAL4/fhRNAi-1). Increased p62 signals. LAMP1GFP immunoreactivity is enhanced nearby the p62 vesicles suggesting the formation of autophagolysosomes to degrade mitochondria. Arrows denote colocalization of LAMP1 and p62 signals. Unfortunately, the reduced size of organelles in glial cells hampered an accurate analysis. (C) Representative GFPATG8a and p62 patterns in GGCs of a 35-day-old control fly (Repo-GAL4/ UAS-GFPATG8a). (D) In glia, besides some cytoplasmisc localitation, GFPATG8a also displayed a clear nuclear pattern as it colocalized with the glial-specific transcription factor Repo (arrows). This nuclear distribution is in agreement with the findings reported for fly GFPATG8a and mammalian LC3/ATG8 (Mauvezin et al., 2014; Huang et al., 2015), respectively). This localization is necessary for the deacetylation of LC3 by Sirt1. Loss of acetyl groups at two specific lysine residues is required for interaction with ATG7 that triggers the formation of autophagosomes (Huang et al., 2015). (E) Quantification of GFPATG8a and LAMPGFP western blots from muscles. Clear increase of Free GFP upon frataxin silencing also in muscles. This result might suggest increased mitophagic flux in muscles too. (F) Representative picture of LAMP1GFP and p62 patterns in 5-day-old control muscles (UAS-LAMP1GFP/+; Mef2-GAL4/UAS-mcherryRNAi). (G) Representative picture of GFPATG8a and p62 patterns in 5-day-old control flies (Mef2-GAL4/UAS-GFPATG8a). Scale bars represent 40 μm in (A–D) and 10 μm in (F,G). Graph in (E) represents means ± SEM. Statistical analysis was performed by Unpaired T-test. *P [file Image_3.TIF]

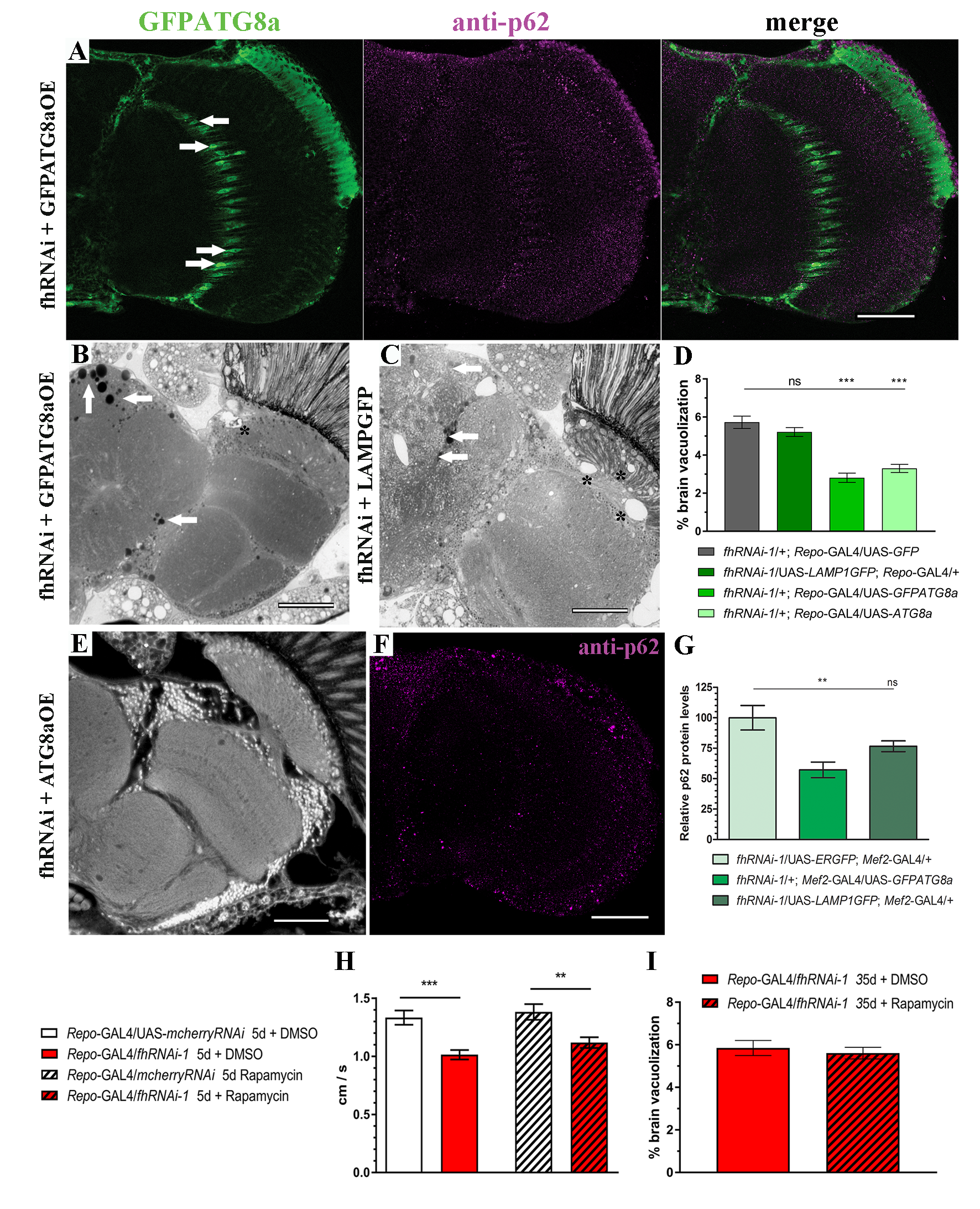

Supplement: FIGURE S4 — Effects of genetic and chemical induction of autophagy in frataxin-deficient flies. (A) Representative GFPATG8a and p62 patterns in the optic lobe of a 35-day-old FRDA fly (fhRNAi-1/+; Repo-GAL4/UAS-GFPATG8a). Interestingly, no p62 accumulation is detected in the GGCs region. Arrows denote autophagosomes. (B) Representative semithin epon plastic section of a 35-day-old frataxin-deficient fly coexpressing GFPATG8a. Brain vacuolization in the lamina and in the outer chiasm was significantly reduced. However, GFPATG8a coexpression was not sufficient to counteract the accumulation of lipid droplets (arrows), another hallmark of frataxin-deficiency in Drosophila glia (Navarro et al., 2010). (C) Representative semithin epon plastic section of a 35-day-old frataxin-deficient fly coexpressing LAMP1GFP. A strong vacuolization (asterisks) is detected in the lamina and in the outer chiasm. The presence of lipid accumulation is denoted by droplets distributed throughout the brain (arrows as example). (D) Quantification of degenerated area (%) in 35-day-old frataxin-deficient flies expressing UAS-LAMP1GFP, UAS-GFPATG8a and UAS-ATG8a compared to coexpression of UAS-GFP analyzed area. Vacuolization was improved upon expression of ATG8a transgenes. (E) Representative paraffin brain section from a 35-day-old fly displaying frataxin knockdown and expression of ATG8a in glial cells (fhRNAi-1/+; Repo-GAL4/UAS-ATG8a). Strong reduction of brain vacuolization was observed. (F) Representative p62 patterns in the optic lobe of a 35-day-old FRDA fly coexpressing UAS-ATG8a. No p62 accumulation was detected in the GGCs region. (G) p62 levels in frataxin-deficient muscles overexpressing autophagy markers GFPATG8a and LAMP1GFP. Muscles with increased expression of GFPATG8a showed lower levels of p62 compared to individuals overexpressing either the lysosomal marker LAMP1GFP or an unrelated marker (ERGFP). (H) Negative locomotor performance of 5-day-old controls and flies displaying glial-specifi [file Image_4.TIF]

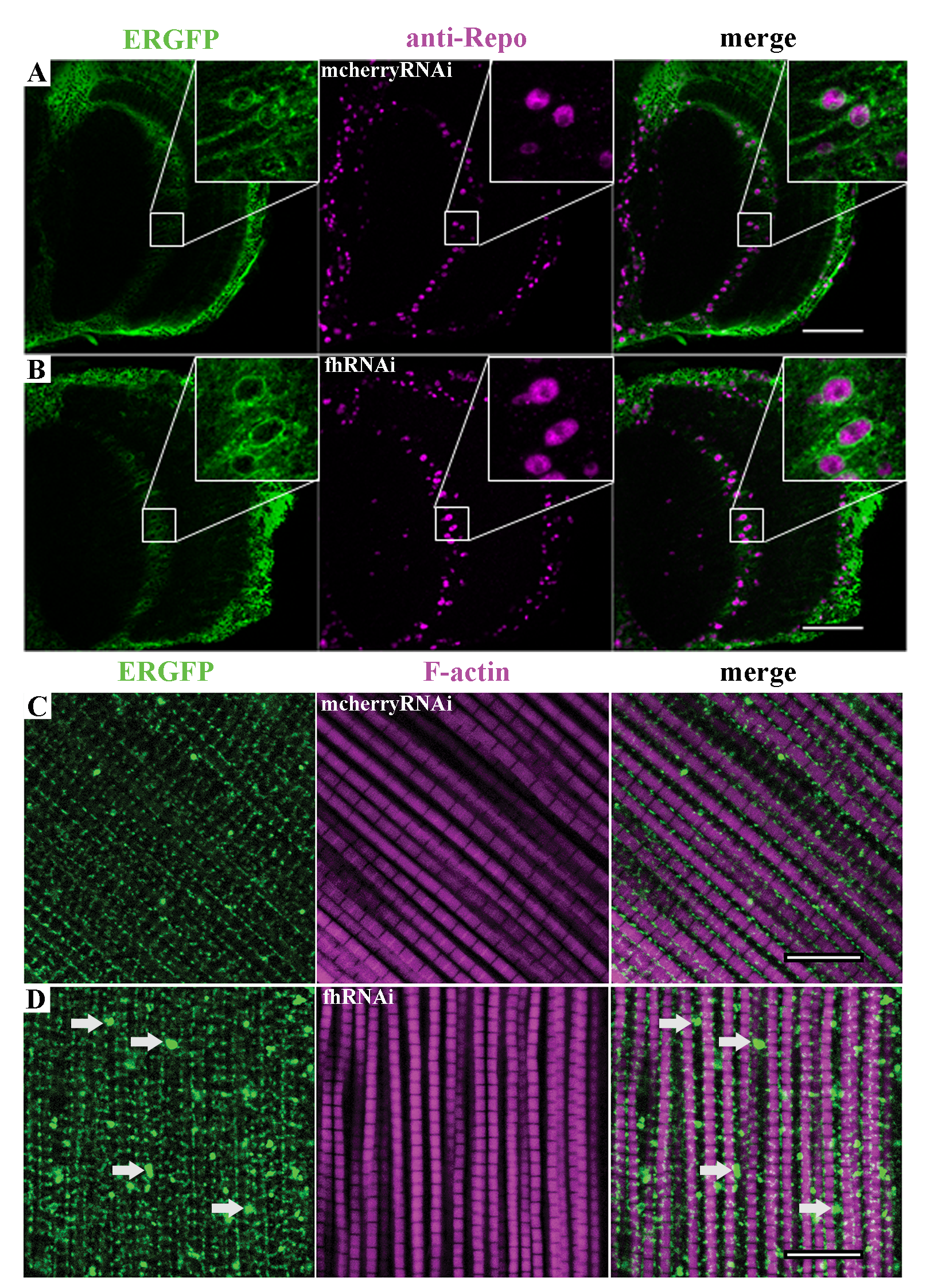

Supplement: FIGURE S5 — ER morphology in frataxin-deficient glial cells and muscles. (A) Representative image of 35-day-old control GGCs (UAS-ERGFP/+; Repo-GAL4/+) stained with anti-Repo antibody to label cell nuclei. Perinuclear localization of ER cisternae is observed. (B) Representative image of 35-day-old frataxin-depleted GGCs (UAS-ERGFP/fhRNAi-1; Repo-GAL4/+) stained with anti-Repo antibody to label cell nuclei. No important alteration of the ER network was detected. (C) Representative image of 7-day-old control muscles (UAS-ERGFP/+; Mef2-GAL4/+) stained with phalloidin to label F-actin of muscle fibers. ER is organized as single signals in a ladder-like network along the muscle fibers with occasional aggregates of ERGFP signals. (D) Representative image of 7-day-old frataxin-depleted muscles (UAS-ERGFP/fhRNAi-1; Mef2-GAL4/+) stained with phalloidin to label F-actin of muscle fibers. The overall organization of ER cisternae is well conserved, although a strong increase of GFP positive clusters is present throughout the muscular system (arrows) disturbing the ER network. Scale bars represent 40 μm in (A,B) and 10 μm (C,D). [file Image_5.TIF]

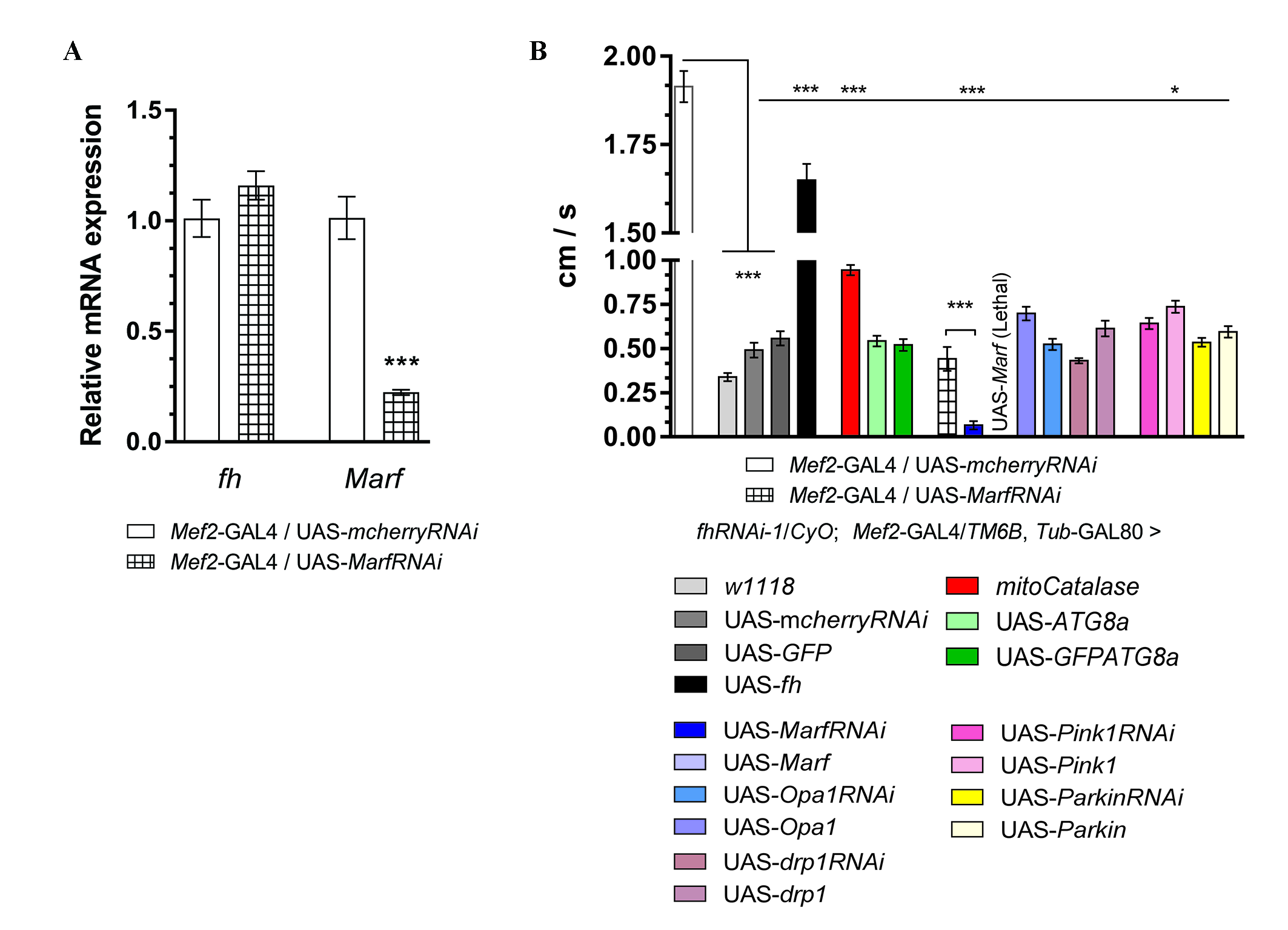

Supplement: FIGURE S6 — Mitofusin silencing fails to improve frataxin deficiency in muscle cells. (A) Marf downregulation does not influence frataxin levels. Since targeted analysis of frataxin levels in glia are methodologically not possible (lack of appropriate antibodies to detect endogenous frataxin or the masking effects of neurons in a Real Time PCR approach), the experiments were performed in fly muscles. (B) Negative geotaxis performance of 5-day-old flies displaying muscle-specific frataxin silencing and coexpression of our transgene’s collection. Besides frataxin overexpression, none of the other potential interactors was able to significantly improve locomotion of FRDA flies. Remarkably, Marf silencing worsened the phenotype. Graphs represent means ± SEM. Data was analyzed by Unpaired T test in (A) and one-way ANOVA with post hoc Dunnett Multiple Comparison Test in (B). *P < 0.05; ***P < 0.001. [file Image_6.TIF]

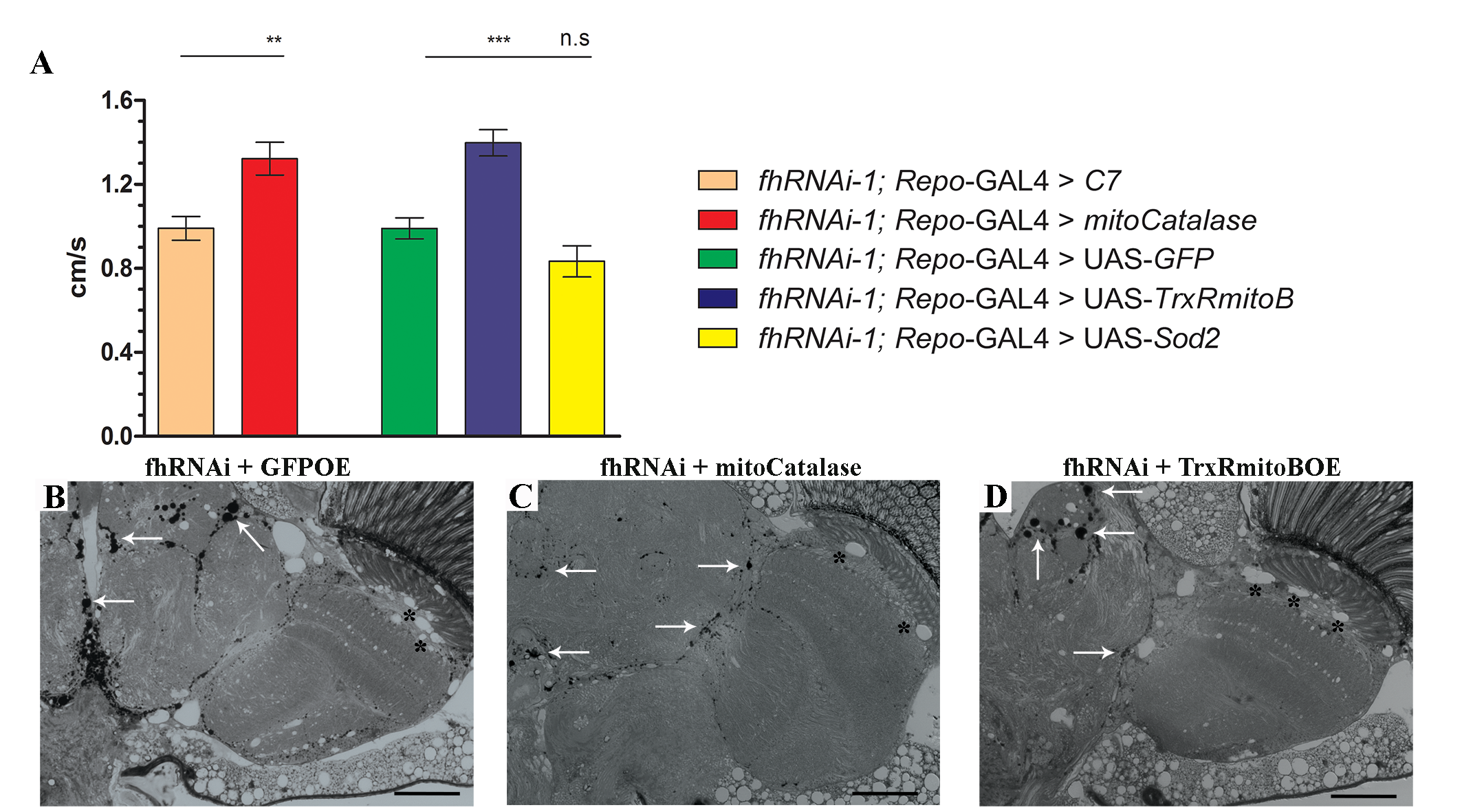

Supplement: FIGURE S7 — Impact of hydrogen peroxide scavengers on FRDA phenotypes. (A) Locomotor performance of 10-day-old flies displaying glial-specific frataxin silencing and coexpression of antioxidant genes. (B–D) Representative ultrathin plastic epon section of a 35-day-old frataxin-deficient flies coexpressing a neutral transgene (B, fhRNAi-1/+; Repo-GAL4/UAS-GFP), mitochondrial catalase (C, fhRNAi-1/+; Repo-GAL4/mitoCatalase) or mitochondrial thioredoxreductase (D, fhRNAi-1/+; Repo-GAL4/UAS-TrxmitoB). No rescue of lipid accumulation (arrows) and brain vacuolization (asterisks) was detected in the two tested genotypes. In (A), means ± SEM, and data was analyzed by one-way ANOVA with post hoc Dunnett Multiple Comparison Test. **P < 0.01; ***P < 0.001. Scale bars represent 50 μm in all cases. [file Image_7.TIF]
